# Mean systolic blood pressure above the control threshold in people with treated uncontrolled hypertension: a pooled, cross-sectional analysis of 55 national health surveys

**DOI:** 10.1016/j.eclinm.2023.101833

**Published:** 2023-01-30

**Authors:** Rodrigo M. Carrillo-Larco, Wilmer Cristobal Guzman-Vilca, Xiaolin Xu, Antonio Bernabe-Ortiz

**Affiliations:** a Department of Epidemiology and Biostatistics, School of Public Health, Imperial College London, London, UK; b Hubert Department of Global Health, Rollins School of Public Health, Emory University, Atlanta, GA, USA; c CRONICAS Centre of Excellence in Chronic Diseases, Universidad Peruana Cayetano Heredia, Lima, Peru; d Universidad Continental, Lima, Peru; e School of Medicine “Alberto Hurtado”, Universidad Peruana Cayetano Heredia, Lima, Peru; f Sociedad Científica de Estudiantes de Medicina Cayetano Heredia (SOCEMCH), Universidad Peruana Cayetano Heredia, Lima, Peru; g Department of Big Data in Health Science School of Public Health, and Center of Clinical Big Data and Analytics of the Second Affiliated Hospital, Zhejiang University School of Medicine, Hangzhou, Zhejiang, China; h School of Public Health, Faculty of Medicine, The University of Queensland, Brisbane, QLD, Australia; i Universidad Cientifica del Sur, Lima, Peru

**Keywords:** Hypertension, Noncommunicable diseases, Global health, Health metrics

## Abstract

**Background:**

The hypertension care cascade has been characterized worldwide, yet it has not been quantified how far above the blood pressure control threshold people with uncontrolled treated hypertension are. We summarized the mean systolic blood pressure (SBP; mmHg) in people treated for hypertension but SBP not <130/80.

**Methods:**

We did a cross-sectional analysis of 55 WHO STEPS Surveys (n = 10,658), comprising six world regions (Africa, Americas, Eastern Mediterranean, Europe, Southeast Asia and Western Pacific); we only included the most recent survey by country regardless of when it was conducted. Adults, men and women, aged between 25 and 69 years, with self-reported hypertension receiving antihypertensive medication and whose blood pressure was >130/80 mmHg were included. We quantified the mean SBP overall and by socio-demographic (sex, age, urban/rural location, education) and cardiometabolic (current smoking, self-reported diabetes) risk factors.

**Findings:**

The lowest SBP was observed in Kuwait (146.6; 95% CI: 143.8–149.4 mmHg) and the highest in Libya (171.9; 95% CI: 167.8–176.0 mmHg). In 29 countries, the SBP was higher in men, and SBP tended to be higher in older groups except in six countries. In 17 countries, the SBP was higher in rural than in urban sites, for example in Turkmenistan the SBP was 162.3 (95% CI: 158.4–166.2) mmHg in rural versus 151.6 (95% CI: 148.7–154.4) mmHg in urban areas. In 25 countries, the SBP was higher in adults with no education, for example in Benin the SBP in people without formal education was 175.3 (95% CI: 168.8–181.9) mmHg versus 156.4 (95% CI: 148.8–164.0) mmHg in people with higher education.

**Interpretation:**

Stronger interventions to improve and secure access to effective management are needed in most countries and specific groups, to reach hypertension control in people with hypertension already receiving antihypertensive medication.

**Funding:**

The 10.13039/100010269Wellcome Trust International Training Fellowship (214185/Z/18/Z).

**Research in context**

**Evidence before this study**

The global evidence on the hypertension care cascade has been well-described including trends since 1990 for all countries in 2021 by the NCD-RisC. The hypertension care cascade comprises three steps: awareness (i.e., people know they have hypertension); treatment (i.e., people with hypertension and receiving antihypertensive medication); and control (i.e., people with hypertension who achieved blood pressure control targets). Nonetheless, knowing the control rate (i.e., proportion of people with hypertension who has achieved blood pressure control thresholds) tells us half of the story, with the other half being: *for those patients with hypertension who have not achieved blood pressure control targets, how far above the target are they?* This information is missing across multiple countries. We searched PubMed on January 4th 2023 using the terms: ((hypertension[tiab] AND ((medication OR treatment) AND control) and blood pressure[tiab]) AND national[tiab]). There were 224 reports, many informing about the hypertension care cascade and its determinants in different national populations. To the best of our knowledge, there were no reports about the gap between the mean blood pressure and the target blood pressure in people with uncontrolled hypertension.

**Added value of this study**

Leveraging on 55 national health surveys in which blood pressure was measured with standard methods, we identified people with self-reported hypertension receiving antihypertensive medication yet whose blood pressure was still above 130/80 mmHg (herein defined as blood pressure control threshold). In such group, we quantified the mean systolic blood pressure and described how far above the control blood pressure threshold the mean was. In so doing, we advanced the global evidence on the hypertension care cascade showing the magnitude of the task ahead to bring people with treated uncontrolled hypertension below the blood pressure control threshold. Arguably, when the goal seems far away, stronger or multiple tasks are needed to reach the goal; conversely, when the goal appears easy to achieve, fewer efforts may suffice to reach the goal. For example, a country where the mean systolic blood pressure in people with treated uncontrolled hypertension was 1 mmHg above the blood pressure control threshold would need, possibly, fewer efforts than a country where the gap is 10 mmHg.

**Implications of all the available evidence**

The global evidence on hypertension care cascade mapped where treatment and control rates were not optimal. Our work advanced this evidence by showing that the task to bring patients with treated uncontrolled hypertension below the control threshold is daunting; we observed that the mean systolic blood pressure was 16 mmHg above the control threshold in the best-case scenario. This work provides a new metric to complement the care cascade, currently only including awareness, treatment, and control.

## Introduction

Despite available treatments and interventions,^1^, ^2^, ^3^ raised blood pressure remains the leading risk factor for cardiovascular diseases which are the leading cause of mortality worldwide.^4^^,^^5^ To lessen the morbidity and mortality burden of raised blood pressure, people with hypertension should receive antihypertensive medication and meet the criteria for blood pressure control (e.g., systolic blood pressure (SBP)/diastolic blood pressure (DBP) <130/80 mmHg^1^). Unfortunately, the antihypertensive treatment coverage and the blood pressure control rates in people with hypertension vary globally,^6^ with several countries showing poor treatment and control rates.^6^ Although strong evidence has characterized the epidemiology of hypertension care cascade globally (i.e., awareness, treatment, and control),^6^ there is no evidence about the magnitude of the task ahead to improve control rates. In other words, there is no evidence on whether the mean blood pressure of people with uncontrolled treated hypertension is 1 mmHg or 10 mmHg higher than the control threshold. This dearth of evidence precludes tailored interventions in terms of magnitude and target population. For example, a country where the blood pressure control threshold has been exceeded by 10 mmHg would need bigger efforts than a country where the mean blood pressure in people with uncontrolled treated hypertension is 1 mmHg higher than the control threshold. Similarly, if the mean blood pressure is 10 mmHg higher than the blood pressure control threshold in men, older people, people in rural sites, or people with low level of education, is largely unknown and could pinpoint populations for tailored interventions. We quantified the mean SBP in people with uncontrolled treated hypertension, to complement and advance the available evidence on hypertension care cascade which has informed about the proportion of people with uncontrolled hypertension worldwide since 1990,^6^ supplementing it with actionable data focusing on SBP which: i) is the leading cause of cardiovascular diseases^4^; ii) has the strongest association with cardiovascular diseases^7^^,^^8^; and iii) is instrumental in clinical trials.^9^^,^^10^ We provide findings for 55 countries and stratified the results by socio-demographic and cardiometabolic risk factors. In so doing, we characterize and map where people with treated hypertension are close or far from the blood pressure control threshold for SBP.

## Methods

### Study design and data sources

Cross-sectional empirical analysis of a pooled dataset of WHO STEPS Surveys.^11^ We only pooled one survey per country, the most recent survey available; also, we only pooled surveys covering a national sample. The WHO STEPS Surveys^11^ follow a consistent sampling procedure and use standard questionnaires and protocols which include measuring blood pressure three times. We adhered to the STROBE reporting guidelines for cross-sectional studies.

We pooled individual-level data of all surveys and excluded observations with implausible values in all three blood pressure measurements (Supplementary Fig. S1): DBP <50 mmHg and >150 mmHg, as well as SBP <70 mmHg and >270 mmHg.^6^ We excluded missing observations in any of the three blood pressure measurements, in self-reported hypertension diagnosis, and self-reported antihypertensive medication. Finally, we excluded missing observations in the variables describing the complex sampling design of each survey (e.g., stratum, primary sampling unit, and sampling weights).

The WHO STEPS Surveys are freely available online through the official data repository. The WHO STEPS Surveys are available for independent reanalysis and do not contain personal identifiers. We deemed this work as of minimal risk and did not seek approval by an Institutional Review Board.

### Study population

In the analysis, we included adults aged between 25 and 69 years (inclusive) because this age band was consistent across surveys (i.e., fewer surveys included younger or older people) (Supplementary Fig. S1). We only included adults with self-reported hypertension, who were taking antihypertensive medication (self-reported through health questionnaires), and whose SBP/DBP was >130/80 mmHg^1^. These selection criteria led to a substantial sample size reduction because we are studying a small fraction of the population (Supplementary Fig. S1): people with hypertension receiving antihypertensive medication and with uncontrolled blood pressure. Thus, the mean SBP is summarized for people with uncontrolled treated hypertension only. To secure enough number of observations for subgroup analyses in each country, we further excluded seven (n = 191) surveys with <40 individuals left.

### Definitions

We summarized the mean SBP at the country level and stratified by socio-demographic determinants and cardiometabolic risk factors. Of note, throughout the manuscript where we refer to mean SBP, such mean is based on the average of the last two of three blood pressure measurements. The results focused on SBP only because the association between SBP and cardiovascular diseases may be stronger than for DBP and cardiovascular diseases^7^^,^^8^; furthermore, SBP has been a key component (e.g., inclusion criteria) of large clinical trials.^9^^,^^10^

We stratified the mean SBP by socio-demographic determinants: i) age, collapsed in three groups (<40, 40–54, and 55+ years); ii) sex (men and women); iii) urban/rural location as collected and defined by each WHO STEPS Survey; and iv) level of education, collected with questionnaires and collapsed in four levels (none, some primary/primary, secondary/high, and university+). We also stratified the mean SBP by cardiometabolic risk factors: i) current smoking, collected with questionnaires (no versus yes); and ii) self-reported diabetes diagnosis, collected with questionnaires (no versus yes).

### Statistical analysis

This is a descriptive analysis conducted with R (version 4.1.2). We summarized the mean SBP at the country level and stratified by all levels of the socio-demographic determinants and cardiometabolic risk factors. The mean SBP is presented alongside the 95% confidence intervals (95% CI). The results account for the complex sampling design of each survey and are therefore informative at the general population level in each country. This is a descriptive work to summarise the mean SBP and we did not aim to claim *statistically* significant differences between groups or countries. Whenever we argue there was a *strong* or *substantial* difference between two groups (e.g., men versus women), it is because the 95% CI did not overlap. Results are presented with figures and the underlying exact numbers are provided as Supplementary Materials. Countries were grouped into geographic regions according to the classification by the World Health Organization; likewise, countries were clustered according to income group based on the World Bank classification.

### Role of the funding source

The funder of the study had no role in study design, data collection, data analysis, data interpretation, or writing of the report. RMC-L and WCG-V had full access to the data and conducted the statistical analyses, and they are the guarantors of the study and vouch for the accuracy of the results. RMC-L had final responsibility for the decision to submit for publication.

## Results

### Study sample

The analysis included surveys from 55 countries and 10,658 records met the selection criteria (Supplementary Table S1). The oldest survey was conducted in 2004 and the most recent survey in 2019; of note, 31/55 (56%) of all surveys were conducted since 2015. One survey (Tokelau; Western Pacific) contributed with 40 people (smallest sample size across all surveys included in the analysis), whilst the largest sample size was from Belarus (Europe; n = 1025). Across all surveys, the mean sample size was 193 (standard deviation: 171.8), and the median sample size was 147 (percentile 25th: 85; percentile 75th: 257). Cook Islands had the smallest proportion of female individuals (Western Pacific; 40.0%), and the largest proportion of women was in Lesotho (Africa; 91.8%). The youngest sample was seen in Bangladesh (Southeast Asia; 49.0 years) and the oldest in Georgia (Europe; 58.7 years).

Overall (Supplementary Table S2), and accounting for the complex sampling design of each survey, the lowest mean SBP in people with uncontrolled treated hypertension at the country level was observed in Kuwait (Eastern Mediterranean; 146.6; 95% CI: 143.8–149.4 mmHg) and the highest in Libya (Eastern Mediterranean; 171.9; 95% CI: 167.8–176.0 mmHg). Highest means were observed in Africa and Europe (Supplementary Fig. S2). There seemed to be gradient whereby lower means were observed in high-income countries and highest means in low-income countries (Supplementary Fig. S3). Of note, results showing the median and the percentile 25th and 75th are available in Supplementary Table S3 revealing largely the same findings.

### Mean systolic blood pressure by socio-demographic determinants

**Fig. 1:**
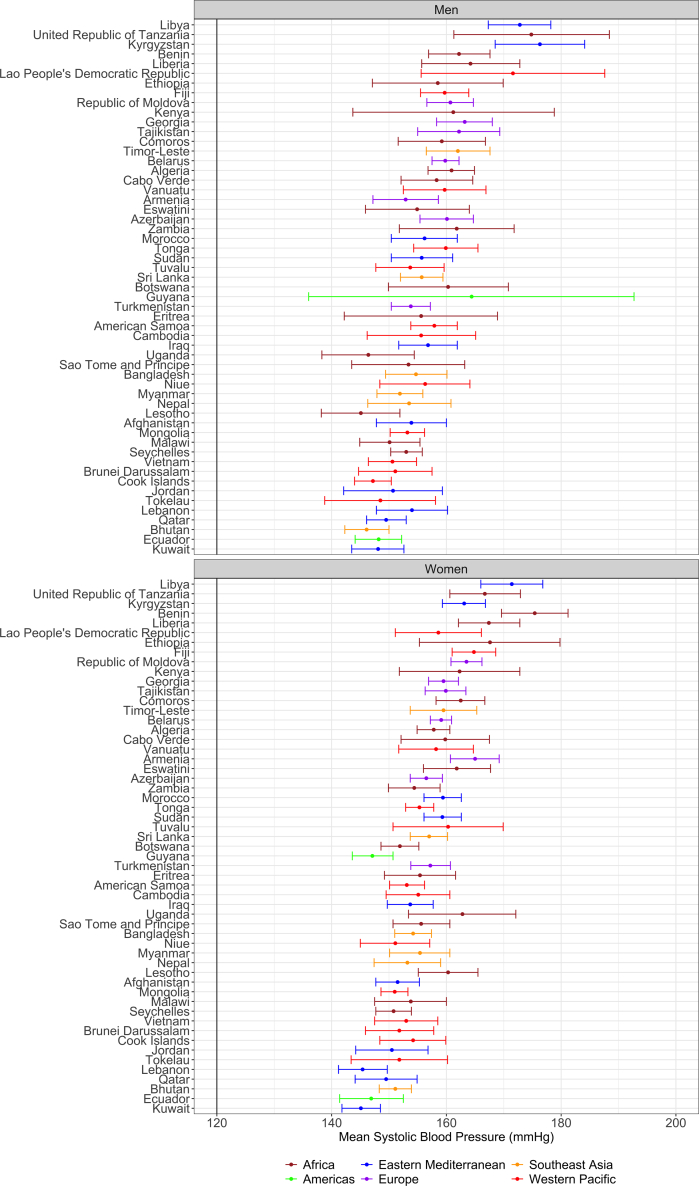
**Mean systolic blood pressure (SBP) by sex and country.** This plot shows the mean (dot) alongside the 95% confidence interval. The underlying results are available in Supplementary Table S4. Countries are presented in ascending order based on their mean SBP in both sexes combined. These results account for the sampling design of each survey.

In 29/55 countries the mean SBP was higher in men than in women, and in Qatar (Eastern Mediterranean) the mean SBP was virtually the same between men (149.5; 95% CI: 146.1–153.0 mmHg) and women (149.5; 95% CI: 144.1–154.9 mmHg). The mean SBP in countries in the Americas was either higher in men or similar between men and women (Fig. 1 and Supplementary Fig. S4); for the other world regions, there was not a clear pattern. The largest women disadvantage was found in Uganda (Africa): 162.8 (95% CI: 153.4–171.1) mmHg in women versus 146.4 (95% CI: 138.3–154.4) mmHg in men. On the other hand, the largest men disadvantage was observed in Guyana (Americas): 164.4 (95% CI: 136.0–192.7) mmHg in men versus 147.1 (95% CI: 143.6–150.7) mmHg in women.

**Fig. 2:**
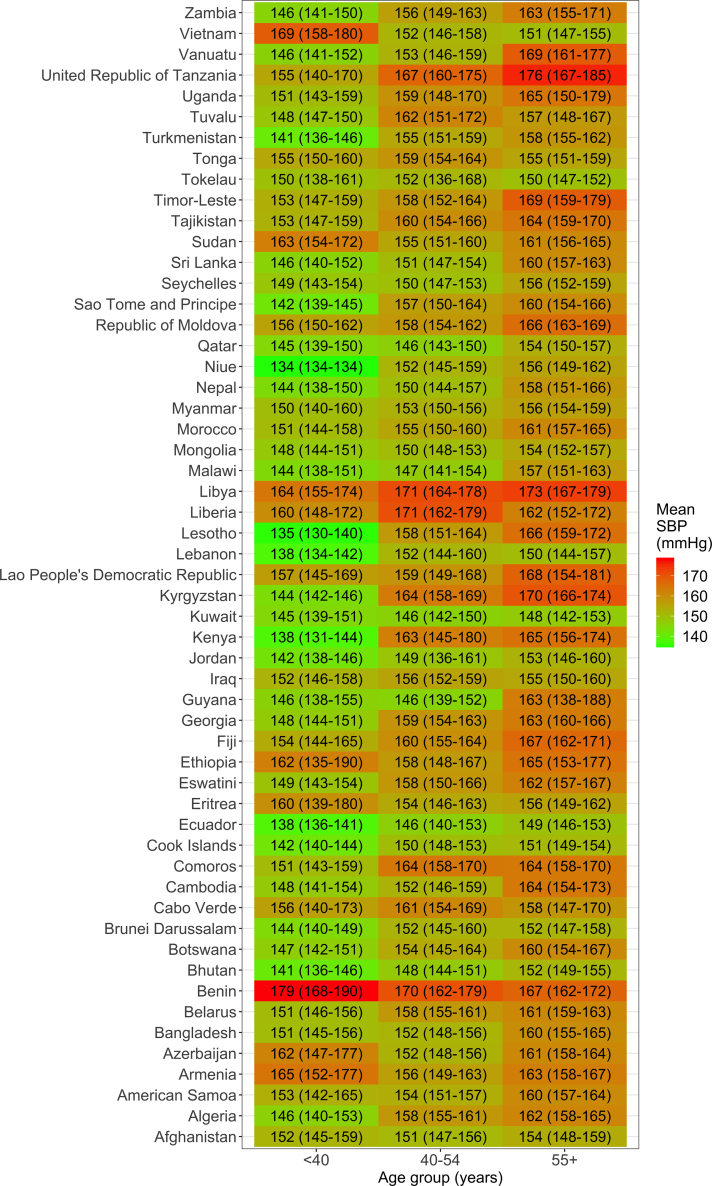
**Mean systolic blood pressure (SBP) by age group and country.** The underlying results are available in Supplementary Table S5. These results account for the sampling design of each survey.

In general, the mean SBP increased with age, yet there were six countries where the opposite pattern was observed (Fig. 2). In Armenia (Europe), Azerbaijan (Europe), Benin (Africa), Eritrea (Africa), Sudan (Eastern Mediterranean), and Vietnam (Western Pacific), the mean SBP seemed to be highest in the youngest age group than in any other age group.

**Fig. 3:**
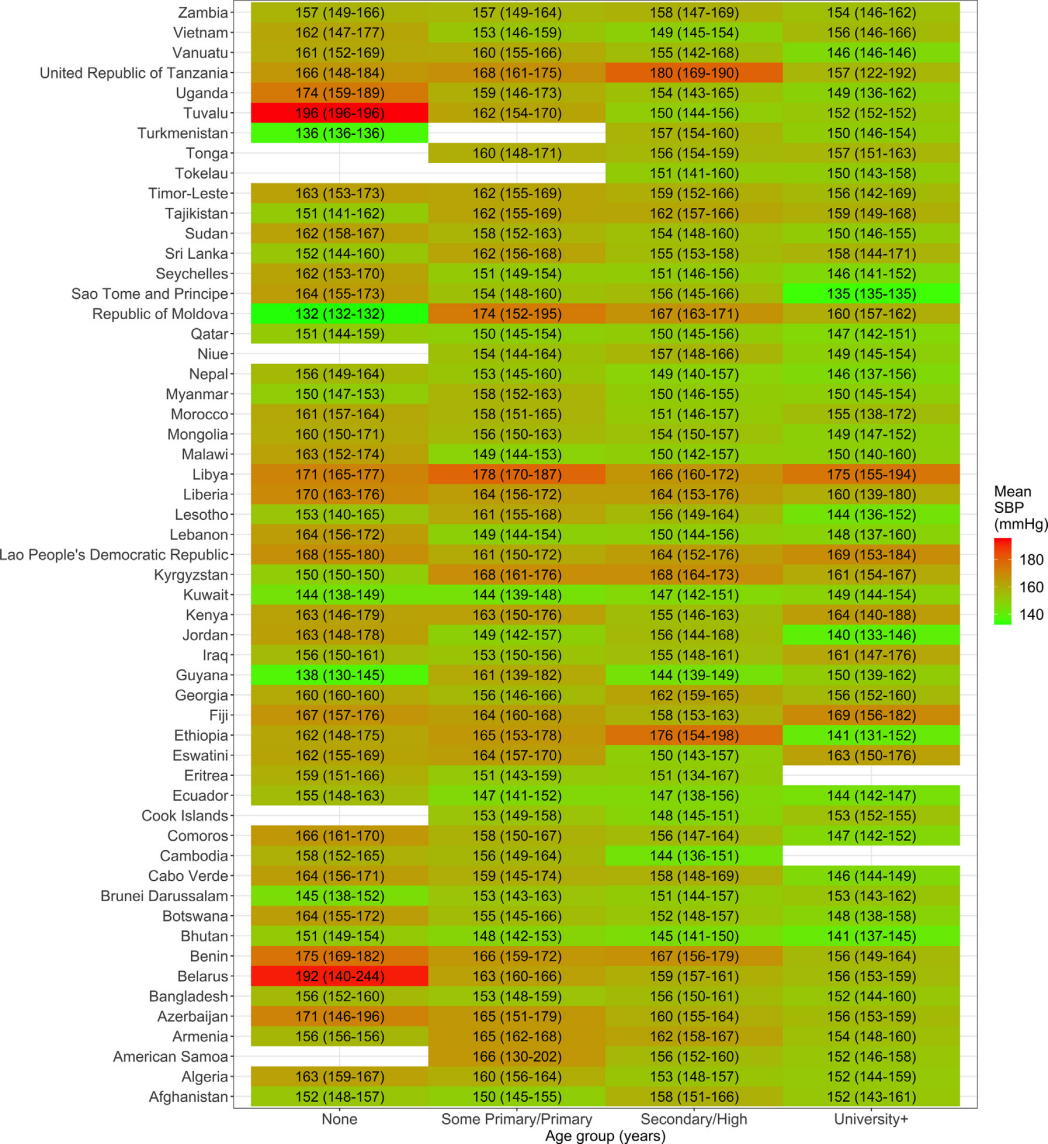
**Mean systolic blood pressure (SBP) by education level and country.** The underlying results are available in Supplementary Table S6. Blank cells because there were no observations in such country-strata. These results account for the sampling design of each survey.

In 25/55 (45%) countries, the mean SBP was highest in adults with no formal education (Fig. 3). Although 95% CI overlapped between the groups based on educational attainment, in 12 countries there was no overlap between the bottom and top groups. In Benin (Africa), for example, the mean SBP in people with no formal education was 175.3 (95% CI: 168.8–181.9) mmHg versus 156.4 (95% CI: 148.8–164.0) mmHg in people with university or higher education.

**Fig. 4:**
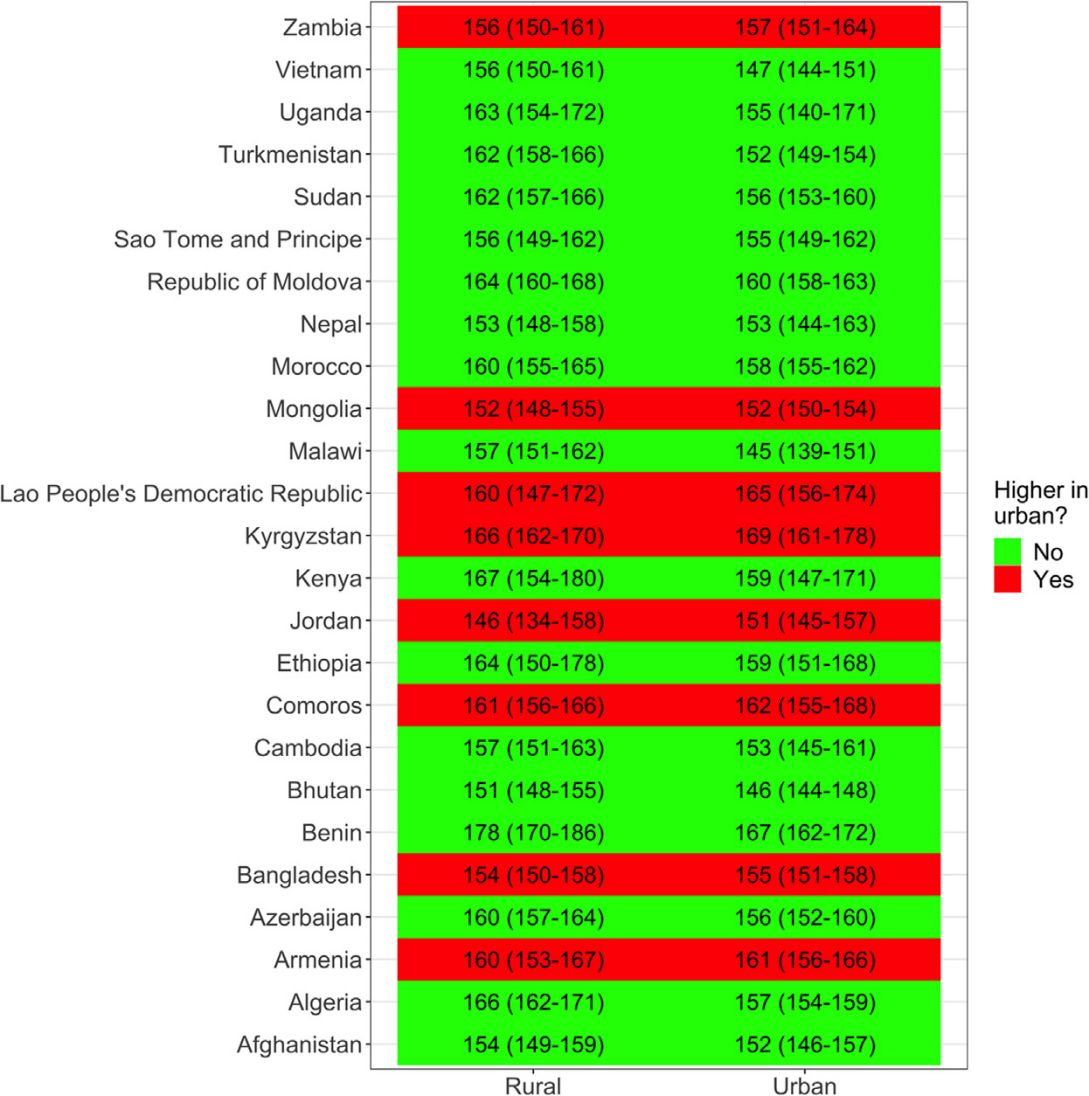
**Mean systolic blood pressure (SBP) by urban or rural location and country.** The underlying results are available in Supplementary Table S7. These results account for the sampling design of each survey.

In 17/25 (68%) countries with urban/rural location information, the mean SBP was higher in rural than in urban sites (Fig. 4). The largest rural disadvantage was found in Turkmenistan (Europe) with a 11 mmHg difference: 162.3 (95% CI: 158.4–166.2) mmHg in rural versus 151.6 (95% CI: 148.7–154.4) mmHg in urban areas. When the mean SBP was higher in urban areas, the 95% CI always overlapped implying there were no strong differences between urban and rural sites.

### Mean systolic blood pressure by cardiometabolic risk factors

**Fig. 5:**
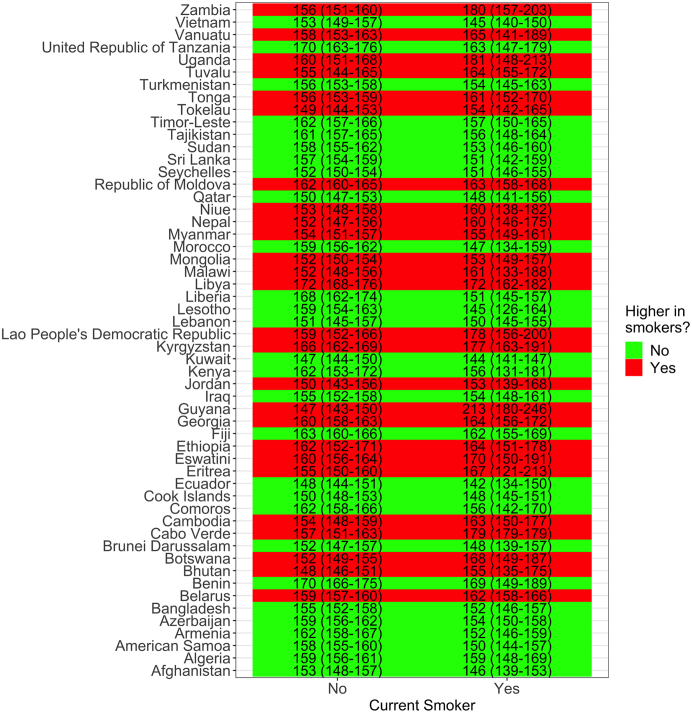
**Mean systolic blood pressure (SBP) by current smoking status and country.** The underlying results are available in Supplementary Table S8. These results account for the sampling design of each survey.

In 26/54 (48%) surveys with information about current smoking status, the mean SBP was higher in people that currently smoked at the time of the survey than in people who did not smoke (Fig. 5). The largest difference was seen in Guyana (Americas): 146.8 (95% CI: 143.4–150.1) mmHg in non-smokers versus 212.7 (95% CI: 179.5–245.9) mmHg in current smokers. When the mean SBP was higher in non-smokers, there were no strong differences (i.e., the 95% CI always overlapped) except in Liberia (Africa) where the mean SBP in non-smokers was 167.8 (95% CI: 161.5–173.9) mmHg and in current smokers the mean SBP was 151.1 (95% CI: 145.0–157.2) mmHg.

**Fig. 6:**
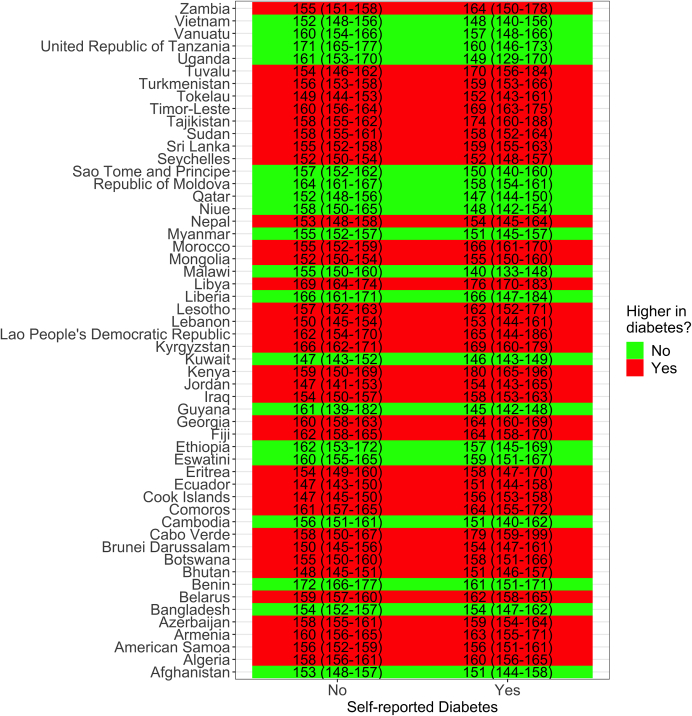
**Mean systolic blood pressure (SBP) by self-reported diabetes status and country.** The underlying results are available in Supplementary Table S9. These results account for the sampling design of each survey.

In 35/54 (65%) surveys with information about self-reported diabetes, the mean SBP was higher in people with self-reported diabetes than in their otherwise healthy counterparts (Fig. 6). The largest difference was observed in Kenya (Africa) where the mean SBP in people without self-reported diabetes was 159.0 (95% CI: 149.5–168.6) mmHg and in people with self-reported diabetes the mean SBP was 180.4 (95% CI: 164.6–196.3) mmHg, though the 95% CI overlapped implying there were no strong differences.

## Discussion

Leveraging on 55 national health surveys we quantified the mean SBP in people with uncontrolled treated hypertension. At the country level, in the best-performing country, the mean SBP was 16 mmHg higher than the SBP control threshold (130 mmHg^1^); conversely, in the worst-performing country the mean SBP was 41 mmHg higher than the SBP control threshold. There was an heterogenous sex pattern whereby in 29/55 countries the mean SBP seemed to be higher in men than in women. As expected, the mean SBP tended to be higher with older age, though there were six countries where the mean SBP appeared to be highest in the youngest age group. In 25/55 countries, the mean SBP seemed to be highest in people with no formal education; similarly, in 17/25 countries, the mean SBP seemed to be higher in people living in rural than urban areas. Finally, in 26/54 countries, the mean SBP appeared to be higher in current smokers, and in 35/54 countries, the mean SBP seemed be higher in people with self-reported diabetes.

These results assist individual countries and global organizations working to improve blood pressure control by quantifying the magnitude of the gap they need to reduce. Previous research has informed about the prevalence of uncontrolled hypertension worldwide, revealing there was a problem (i.e., plenty of people with hypertension above the control threshold).^6^ Our work advances this evidence by quantifying how far above the control threshold people with uncontrolled hypertension are (i.e., gap between the observed mean blood pressure and the control threshold). Individual countries and global organizations can use this information to propose short- and long-term plans. For example, countries where the gap is small can aim for a short-term aggressive plan to improve hypertension control rates. On the other hand, countries where the gap is substantial, can aim for a long-term plan to reduce the gap in sequential steps. Moreover, our results can also be useful as baseline to inform intermediate milestones in the path to improve hypertension control rates. That is, increasing the proportion of people with hypertension under the control threshold can be the final target, yet in the meantime countries and global organizations can monitor the gap to ascertain whether they are on track to improve hypertension control rates. In other words, if the gap has stagnated or increased, they may not be on track to achieve improvement in hypertension control rates.

We acknowledge that the blood pressure control threshold (130/80 mmHg^1^) we used to define the study population may be seen as an aggressive target, which is difficult to achieve even in high-income countries with solid health systems.^6^ Nonetheless, our choice is still supported by evidence-based clinical guidelines^1^ and goes in line with evidence showing health benefits with aggressive blood pressure targets.^9^ Lastly, we also chose the 130/80 mmHg threshold to maximize our sample size. That is, had we chosen a more lenient threshold (e.g., 140/90 mmHg), the sample size would have been smaller further compromising our results.

Globally, hypertension control rates are usually lower in men than in women.^6^ This is consistent with our results showing that, in most countries, the gap between the SBP control threshold and the mean SBP was larger in men. Potential explanations include regular contact with healthcare providers and better adherence to antihypertensive treatment in women.^12^^,^^13^ While previous evidence has suggested better antihypertensive treatment and blood pressure control rates with older age,^6^^,^^12^^,^^13^ our results signalled that the gap between the mean SBP and the blood pressure control threshold was larger with older age. This may suggest that reporting the treatment and control rates alone,^6^^,^^13^ may not tell the full story about the hypertension care cascade which can be complemented with the metric herein presented (i.e., gap in relation to the blood pressure control threshold).

There are no global urban-rural comparisons of the full hypertension care cascade,^14^ nor global comparisons by level of educational. However, several studies have reported worse hypertension metrics in people facing socio-economic difficulties.^12^, ^13^, ^14^, ^15^ Our results showed large gaps between the SBP control threshold and the mean SBP in rural sites. This could be due to limited access to healthcare facilities, and where healthcare facilities are available and accessible, these may not have antihypertensive medications^16^ (even though antihypertensive drugs are in the global list of essential medicines^17^). Furthermore, shortage of physicians^18^ in rural areas to prescribe and titrate the antihypertensive medication could also explain these findings. Affordability^16^ and out-of-pocket as well as catastrophic expenses experienced by people living in rural areas and people with low level of education must be considered too.

We observed larger gaps between the SBP control threshold and the mean SBP in current smokers and people with self-reported diabetes. The relationship between these cardiometabolic risk factors and mean SBP in people with uncontrolled hypertension could be confounded by education and urban/rural location. Disentangling the complex relationship between these variables is beyond the scope of our work. Nevertheless, our results signal missed opportunities to improve blood pressure in people with diabetes and smokers. People with diabetes should regularly visit their healthcare provider; similarly, smokers may receive treatment or counselling to quit smoking. In either case, they should have additional opportunities to have their blood pressure measured and antihypertensive medication prescribed.

Arguably, the most effective intervention to reduce blood pressure in people with hypertension is pharmacological treatment in the context of healthy lifestyles.^1^^,^^2^ Our results show that procuring effective antihypertensive treatment for people with uncontrolled treated hypertension must be improved, particularly in older adults, people with low level of education and living in rural area, as well as in people with diabetes and current smokers. For example, this could be achieved via few routes. First, securing the availability and affordability^16^ of antihypertensive medications should be a priority for all health systems, together with securing the availability of trained healthcare and non-healthcare (e.g., community health workers^19^) personnel to prescribe and titrate such medication. Second, following evidence-based guidelines may facilitate the prescription and monitoring of antihypertensive medication. An outstanding example is the work by the Pan America Health Organization (PAHO) and their *Hypertension Clinical Pathway* being implemented across the region in the context of the HEARTS program.^20^, ^21^, ^22^, ^23^ This sort of programs requires strong commitment by national governments and global health organizations, which could leverage on the recommendations by the global HEARTS Technical Package.^24^ Finally, while clinicians may not directly use our results based on means at the population level, clinicians are instrumental in promoting public health policies. For example, clinicians can take our findings, together with global evidence about the hypertension care cascade,^6^^,^^13^ to advocate to secure the availability of antihypertensive medication and the resources to promote nonpharmacological care for hypertension in their facilities.

Together with the worldwide evidence on hypertension care cascade,^6^^,^^13^ our results suggest that it may not be enough to simplify controlled hypertension in a dichotomous metric (i.e., controlled/uncontrolled). In people with uncontrolled hypertension, despite reporting antihypertensive treatment, monitoring how far above the control threshold they are may help to prioritize settings and subgroups, and to find the best interventions. For example, securing polypills with quadruple^25^^,^^26^ combinations for groups whose blood pressure is far above the control threshold, dual^27^ combinations for groups in the mid-range above the blood pressure control threshold, and perhaps to strengthen their current treatment plus non-pharmacological interventions (e.g., salt reduction^28^^,^^29^) for people close to the blood pressure control threshold.

While our descriptive work to characterize and map whether people with uncontrolled treated hypertension are close or far from the blood pressure control threshold provides some answers, our results also open questions. For example, where the gaps are largest, would interventions with polypills be preferred and cost-effective? Would the available workforce, including both health professional and lay personnel (e.g., community health workers) be enough to improve treatment for people with uncontrolled yet treated hypertension? What new interventions to boost antihypertensive medication adherence are warranted in diverse settings and populations? These questions deserve careful consideration by high-quality research.

Our work has several strengths. We analysed individual-level data from 55 surveys conducted in a national sample. Our results for blood pressure were based on the gold standard: the average of the last two out of three measurements. Moreover, the results accounted for the complex sampling design of each survey. Altogether, our results provide strong evidence to inform policies, strategies, and interventions to improve blood pressure control in people with uncontrolled treated hypertension in 55 countries across the globe.

Notwithstanding, there are limitations we must highlight to improve the interpretation of the methods and results. First, for some countries there was a rather small sample size because we studied a fraction of the population (i.e., people with hypertension receiving antihypertensive medication and whose SBP/DBP was >130/80 mmHg^1^). In this line, the sample size for some countries included much more women than men or vice versa. The results should be interpreted accounting for this sex imbalance. As such, using our results deserve careful consideration examining the sample size, sex-imbalance, and year when they survey was conducted. The small sample size in some countries may compromise the representativeness of the results for the underlying general population in the country; however, the results may still be informative for the underlying population. Future work should verify our findings with a much larger sample size, possibly by pooling several surveys per country or by using national registries of patients with hypertension. Second, because of data availability we included few socio-demographic determinants and cardiometabolic risk factors. Had we included other cardiometabolic risk factors, such as obesity or high cholesterol, the sample size would have been smaller (after accounting for missing observations in these variables and because blood biomarkers were collected in some WHO STEPS Surveys only). Third, this is a descriptive analysis whereby we summarized the mean SBP in a relevant population (people with uncontrolled treated hypertension) stratified by key socio-demographic and clinical determinants. Analysing the association (e.g., odds ratio) between the socio-demographic determinants or cardiometabolic risk factors with SBP was beyond the scope of this work. This, because the role of these socio-demographic determinants and cardiometabolic risk factors in the epidemiology of hypertension has been well studied in the past.^14^^,^^15^^,^^30^, ^31^, ^32^ However, there was no evidence about how far above the threshold of SBP control people with uncontrolled treated hypertension were. Fourth, we did not test any hypotheses to ascertain *statistically* significant differences between countries or groups (e.g., t-tests or ANOVA with Bonferroni post-hoc adjustment for multiple comparisons). The reason was because we aimed to characterize the mean SBP in people with uncontrolled treated hypertension in diverse populations, rather than to claim the mean SBP was *statistically* higher/lower in one country than the other(s). The results without statistical comparisons provide solid evidence for policy makers and public health officers in these 55 countries, who now have data to support the formulation of stronger interventions to achieve substantial SBP reductions overall and in some subgroups. Moreover, from a presentation standpoint, comparisons across countries will deliver 1486 p-values (1 p-value for the ANOVA and 1485 p-values for all possible pair-wise comparisons between 55 countries). These p-values would need to be summarized in a very busy table compromising its readability, interpretability, and appeal to the readers. Fifth, we used self-reported information about antihypertensive treatment which did not incorporate evidence about adherence as it is common practice in global analyses.^6^ This lack of precision regarding adherence could have influenced the results yet it is difficult to ascertain the direction and magnitude of such a bias. Arguably, because of desirability bias, people with hypertension could have reported taking antihypertensive medication even though they were not taking the medication exactly as prescribed (i.e., low adherence). If so, our results could be depicting a worse scenario that it really is. Sixth, information about the exact antihypertensive drug(s) prescribed and taken by the participants was not available. Seventh, this is a cross-sectional analysis of 55 countries only. Future work should complement our results with time series analyses to find whether there have been improvements over the years; similarly, future work should provide this evidence for more or all countries in the world. Eight, the time span covered by the surveys we analysed was long (between 2004 and 2019). It may be the case that the results reported for the earliest surveys do not longer apply to the present time. Furthermore, hypertension guidelines have been updated since 2004 and trials conducted since 2004 may have changed the therapeutic approach in each country. Thus, the results should be interpreted for each country and year. Despite this limitation, our earliest results can serve as benchmark or baseline, and future surveys in these countries can assess whether they have reduced the gap, or the gap has widened. Finally, while the means reported accounted for the sampling design of each survey, the means were not adjusted by the health determinants used to stratify the results (e.g., sex-specific means adjusted by age). Consequently, in line with our descriptive analysis plan, the means are crude and can be confounded by other factors.

The global hypertension care cascade has been characterized,^6^^,^^13^ though evidence was missing on the gap between the SBP control threshold and the mean SBP in people with uncontrolled treated hypertension. In the best-case scenario across 55 countries, the mean SBP in people with uncontrolled treated hypertension was 16 mmHg above the control threshold of 130 mmHg. This gap was larger in rural areas and in people with low formal education, as well as in people with diabetes and current smokers. Stronger interventions to improve and secure access to effective management of hypertension are needed in most countries and specific groups, to reach hypertension control in people with already receiving antihypertensive medication.

## Contributors

RMC-L conceived the idea. RMC-L and WCG-V downloaded and pooled the data. RMC-L and WCG-V conducted the statistical analysis and verified the underlying data. RMC-L and ABO drafted the manuscript with support from all co-authors. All co-authors edited and provided critical input to improve the manuscript through several rounds of revisions. All authors approved the submitted version.

## Data sharing statement

Data can be freely accessed and downloaded from the World Health Organization NCD Repository at https://extranet.who.int/ncdsmicrodata/index.php/home. Access to the data requires personal login credentials, we therefore cannot share the data with the manuscript.

## Declaration of interests

All authors declare no competing interests.
