# Supplementary material for: Mean systolic blood pressure above the control threshold in people with treated uncontrolled hypertension: a pooled, cross-sectional analysis of 55 national health surveys
Source: eClinicalMedicine. 2023 Jan 30;57:101833. doi: 10.1016/j.eclinm.2023.101833 (PMC9923190; doi:10.1016/j.eclinm.2023.101833)
Supplement: Supplementary Tables and Figures [file mmc1.pdf]

Mean systolic blood pressure above the control threshold in people with treated uncontrolled hypertension: a pooled, cross-sectional analysis of 55 national health surveys

Corresponding Author

Rodrigo M Carrillo-Larco, MD, PhD

Department of Epidemiology and Biostatistics,

School of Public Health,

Imperial College London, London, UK.

+44 7578240395

r.carrillo-larco@imperial.ac.uk

Supplementary Table 1. Description of the study population by survey.

DBP: diastolic blood pressure (mmHg); SBP: systolic blood pressure (mmHg). These summaries do not account for the complex sampling design of each survey, because they aim to describe the sample included in the analysis rather than providing summaries representative at the country level.

	Afghanistan	Algeria	American.Samoa	Armenia	Azerbaijan	Bangladesh	Belarus	Benin	Bhutan	Botswana	Brunei Darussalam	Cabo.Verde	Cambodia	Comoros
	(N=257)	(N=262)	(N=109)	(N=167)	(N=292)	(N=319)	(N=1025)	(N=111)	(N=257)	(N=277)	(N=178)	(N=80)	(N=90)	(N=168)
**Age**														
**Mean (SD)**	50.3 (11.5)	55.6 (8.94)	52.1 (7.64)	57.3 (8.27)	56.7 (8.40)	49.0 (9.92)	57.3 (8.54)	52.1 (10.8)	53.9 (10.3)	50.6 (10.3)	53.8 (9.36)	51.5 (8.36)	51.4 (8.10)	49.8 (9.64)
**Median [Min, Max]**	50.0 [25.0, 69.0]	56.0 [25.0, 69.0]	53.0 [27.0, 64.0]	59.0 [26.0, 69.0]	58.0 [29.0, 69.0]	49.0 [25.0, 69.0]	59.0 [26.0, 69.0]	53.0 [27.0, 69.0]	55.0 [27.0, 69.0]	52.0 [25.0, 69.0]	54.0 [25.0, 69.0]	52.0 [29.0, 64.0]	52.5 [27.0, 64.0]	50.0 [26.0, 64.0]
**Age groups**														
**<40**	42 (16.3%)	13 (5.0%)	8 (7.3%)	5 (3.0%)	12 (4.1%)	58 (18.2%)	43 (4.2%)	17 (15.3%)	27 (10.5%)	48 (17.3%)	13 (7.3%)	6 (7.5%)	8 (8.9%)	26 (15.5%)
**40-54**	115 (44.7%)	102 (38.9%)	61 (56.0%)	48 (28.7%)	81 (27.7%)	158 (49.5%)	282 (27.5%)	41 (36.9%)	95 (37.0%)	115 (41.5%)	81 (45.5%)	40 (50.0%)	49 (54.4%)	79 (47.0%)
**55 and above**	100 (38.9%)	147 (56.1%)	40 (36.7%)	114 (68.3%)	199 (68.2%)	103 (32.3%)	700 (68.3%)	53 (47.7%)	135 (52.5%)	114 (41.2%)	84 (47.2%)	34 (42.5%)	33 (36.7%)	63 (37.5%)
**Sex**														
**Men**	98 (38.1%)	85 (32.4%)	60 (55.0%)	27 (16.2%)	96 (32.9%)	112 (35.1%)	326 (31.8%)	41 (36.9%)	90 (35.0%)	64 (23.1%)	90 (50.6%)	17 (21.3%)	21 (23.3%)	28 (16.7%)
**Women**	159 (61.9%)	177 (67.6%)	49 (45.0%)	140 (83.8%)	196 (67.1%)	207 (64.9%)	699 (68.2%)	70 (63.1%)	167 (65.0%)	213 (76.9%)	88 (49.4%)	63 (78.8%)	69 (76.7%)	140 (83.3%)
**SBP**														
**Mean (SD)**	154 (17.1)	159 (19.5)	157 (18.4)	162 (21.5)	158 (18.9)	154 (19.7)	160 (20.3)	168 (22.8)	150 (16.6)	155 (19.7)	149 (17.2)	161 (18.8)	155 (20.9)	163 (22.3)
**Median [Min, Max]**	151 [131, 209]	156 [131, 227]	152 [133, 231]	158 [131, 238]	154 [131, 214]	150 [131, 236]	155 [131, 257]	167 [133, 229]	146 [131, 210]	151 [131, 223]	145 [131, 220]	160 [132, 218]	152 [131, 226]	160 [131, 225]
**DBP**														
**Mean (SD)**	96.4 (10.4)	90.7 (8.61)	97.3 (10.1)	99.3 (10.7)	95.4 (9.60)	97.7 (10.8)	96.9 (10.2)	103 (11.5)	96.8 (9.66)	95.8 (9.83)	92.2 (10.1)	95.7 (9.47)	94.4 (10.5)	98.2 (11.3)
**Median [Min, Max]**	94.5 [80.5, 139]	89.5 [80.5, 130]	96.0 [81.0, 125]	97.5 [81.5, 140]	94.5 [80.5, 133]	97.0 [81.0, 147]	95.0 [80.5, 147]	102 [81.5, 143]	95.5 [80.5, 137]	94.5 [80.5, 136]	90.0 [80.5, 138]	94.8 [80.5, 120]	92.5 [80.5, 138]	96.8 [80.5, 129]
**Location**														
**Rural**	115 (44.7%)	62 (23.7%)	0 (0%)	45 (26.9%)	137 (46.9%)	119 (37.3%)	0 (0%)	44 (39.6%)	161 (62.6%)	0 (0%)	0 (0%)	0 (0%)	54 (60.0%)	92 (54.8%)
**Urban**	142 (55.3%)	200 (76.3%)	0 (0%)	122 (73.1%)	155 (53.1%)	200 (62.7%)	0 (0%)	67 (60.4%)	96 (37.4%)	0 (0%)	0 (0%)	0 (0%)	36 (40.0%)	76 (45.2%)
**Missing**	0 (0%)	0 (0%)	109 (100%)	0 (0%)	0 (0%)	0 (0%)	1025 (100%)	0 (0%)	0 (0%)	277 (100%)	178 (100%)	80 (100%)	0 (0%)	0 (0%)
**Level of education**														
**None**	181 (70.4%)	97 (37.0%)	0 (0%)	1 (0.6%)	3 (1.0%)	92 (28.8%)	2 (0.2%)	59 (53.2%)	167 (65.0%)	57 (20.6%)	7 (3.9%)	29 (36.3%)	29 (32.2%)	101 (60.1%)
**Some Primary/Primary**	30 (11.7%)	91 (34.7%)	4 (3.7%)	2 (1.2%)	9 (3.1%)	132 (41.4%)	265 (25.9%)	29 (26.1%)	37 (14.4%)	113 (40.8%)	35 (19.7%)	40 (50.0%)	50 (55.6%)	34 (20.2%)
**Secondary/High**	30 (11.7%)	44 (16.8%)	80 (73.4%)	134 (80.2%)	131 (44.9%)	65 (20.4%)	563 (54.9%)	14 (12.6%)	28 (10.9%)	94 (33.9%)	93 (52.2%)	8 (10.0%)	11 (12.2%)	30 (17.9%)
**University+**	7 (2.7%)	19 (7.3%)	22 (20.2%)	30 (18.0%)	148 (50.7%)	29 (9.1%)	193 (18.8%)	9 (8.1%)	18 (7.0%)	13 (4.7%)	40 (22.5%)	3 (3.8%)	0 (0%)	2 (1.2%)
**Missing**	9 (3.5%)	11 (4.2%)	3 (2.8%)	0 (0%)	1 (0.3%)	1 (0.3%)	2 (0.2%)	0 (0%)	7 (2.7%)	0 (0%)	3 (1.7%)	0 (0%)	0 (0%)	1 (0.6%)
**Current smoker**														
**No**	241 (93.8%)	250 (95.4%)	84 (77.1%)	153 (91.6%)	251 (86.0%)	277 (86.8%)	859 (83.8%)	106 (95.5%)	251 (97.7%)	250 (90.3%)	161 (90.4%)	77 (96.3%)	78 (86.7%)	157 (93.5%)
**Yes**	16 (6.2%)	12 (4.6%)	25 (22.9%)	14 (8.4%)	41 (14.0%)	42 (13.2%)	166 (16.2%)	5 (4.5%)	6 (2.3%)	27 (9.7%)	17 (9.6%)	3 (3.8%)	12 (13.3%)	11 (6.5%)
**Missing**	0 (0%)	0 (0%)	0 (0%)	0 (0%)	0 (0%)	0 (0%)	0 (0%)	0 (0%)	0 (0%)	0 (0%)	0 (0%)	0 (0%)	0 (0%)	0 (0%)
**Self-reported diabetes**														
**No**	202 (78.6%)	173 (66.0%)	65 (59.6%)	130 (77.8%)	231 (79.1%)	251 (78.7%)	853 (83.2%)	97 (87.4%)	218 (84.8%)	237 (85.6%)	126 (70.8%)	71 (88.8%)	74 (82.2%)	142 (84.5%)
**Yes**	55 (21.4%)	89 (34.0%)	44 (40.4%)	37 (22.2%)	61 (20.9%)	68 (21.3%)	172 (16.8%)	14 (12.6%)	39 (15.2%)	40 (14.4%)	52 (29.2%)	9 (11.3%)	16 (17.8%)	26 (15.5%)
**Missing**	0 (0%)	0 (0%)	0 (0%)	0 (0%)	0 (0%)	0 (0%)	0 (0%)	0 (0%)	0 (0%)	0 (0%)	0 (0%)	0 (0%)	0 (0%)	0 (0%)

Cook Islands	Ecuador	Eritrea	Eswatini	Ethiopia	Fiji	Georgia	Guyana	Iraq	Jordan	Kenya	Kuwait	Kyrgyzstan	Lao People’s Democratic Republic	Lebanon
(N=65)	(N=147)	(N=82)	(N=163)	(N=85)	(N=174)	(N=498)	(N=85)	(N=90)	(N=91)	(N=74)	(N=159)	(N=273)	(N=60)	(N=70)
52.6 (8.08)	55.4 (9.61)	53.9 (9.50)	56.7 (8.60)	51.1 (10.5)	53.4 (7.39)	58.7 (7.95)	54.8 (9.08)	53.2 (9.68)	58.6 (8.95)	54.8 (9.08)	51.4 (9.05)	55.2 (6.52)	51.3 (8.48)	55.2 (7.22)
54.0 [29.0, 64.0]	58.0 [30.0, 69.0]	55.0 [31.0, 68.0]	58.0 [32.0, 69.0]	52.0 [25.0, 69.0]	53.5 [34.0, 64.0]	60.0 [25.0, 69.0]	56.0 [29.0, 69.0]	54.5 [32.0, 69.0]	60.0 [35.0, 69.0]	56.0 [33.0, 67.0]	51.0 [30.0, 69.0]	56.0 [33.0, 64.0]	53.0 [30.0, 64.0]	55.0 [34.0, 68.0]
5 (7.7%)	8 (5.4%)	5 (6.1%)	7 (4.3%)	11 (12.9%)	8 (4.6%)	10 (2.0%)	6 (7.1%)	12 (13.3%)	5 (5.5%)	5 (6.8%)	18 (11.3%)	3 (1.1%)	7 (11.7%)	3 (4.3%)
29 (44.6%)	54 (36.7%)	33 (40.2%)	52 (31.9%)	36 (42.4%)	86 (49.4%)	131 (26.3%)	27 (31.8%)	33 (36.7%)	22 (24.2%)	26 (35.1%)	80 (50.3%)	107 (39.2%)	26 (43.3%)	29 (41.4%)
31 (47.7%)	85 (57.8%)	44 (53.7%)	104 (63.8%)	38 (44.7%)	80 (46.0%)	357 (71.7%)	52 (61.2%)	45 (50.0%)	64 (70.3%)	43 (58.1%)	61 (38.4%)	163 (59.7%)	27 (45.0%)	38 (54.3%)
39 (60.0%)	62 (42.2%)	20 (24.4%)	27 (16.6%)	33 (38.8%)	61 (35.1%)	104 (20.9%)	30 (35.3%)	25 (27.8%)	45 (49.5%)	18 (24.3%)	66 (41.5%)	56 (20.5%)	18 (30.0%)	34 (48.6%)
26 (40.0%)	85 (57.8%)	62 (75.6%)	136 (83.4%)	52 (61.2%)	113 (64.9%)	394 (79.1%)	55 (64.7%)	65 (72.2%)	46 (50.5%)	56 (75.7%)	93 (58.5%)	217 (79.5%)	42 (70.0%)	36 (51.4%)
150 (15.0)	148 (16.4)	157 (23.6)	161 (23.2)	163 (23.5)	163 (20.4)	161 (20.8)	151 (16.2)	155 (13.6)	150 (16.9)	159 (26.2)	146 (13.4)	165 (22.6)	161 (22.8)	149 (14.7)
147 [131, 191]	143 [131, 221]	152 [131, 211]	159 [132, 243]	157 [132, 251]	158 [131, 222]	158 [131, 238]	149 [131, 230]	153 [135, 196]	146 [131, 200]	153 [131, 259]	143 [131, 240]	161 [131, 240]	158 [132, 225]	145 [131, 193]
93.3 (8.47)	92.5 (8.99)	93.5 (10.7)	99.4 (12.2)	99.1 (12.9)	96.1 (10.9)	96.9 (10.4)	94.1 (9.92)	95.7 (10.2)	93.5 (8.23)	97.8 (13.5)	93.5 (7.78)	102 (11.9)	98.2 (10.1)	91.0 (7.77)
91.5 [81.5, 119]	91.5 [80.5, 130]	91.0 [81.0, 130]	97.0 [80.5, 133]	96.5 [81.5, 134]	95.0 [80.5, 131]	96.0 [80.5, 150]	91.5 [80.5, 129]	91.0 [81.0, 145]	92.5 [80.5, 118]	95.5 [80.5, 146]	91.5 [81.0, 124]	100 [80.5, 146]	97.0 [81.0, 123]	89.8 [80.5, 110]
0 (0%)	0 (0%)	0 (0%)	0 (0%)	28 (32.9%)	0 (0%)	0 (0%)	0 (0%)	0 (0%)	11 (12.1%)	38 (51.4%)	0 (0%)	170 (62.3%)	30 (50.0%)	0 (0%)
0 (0%)	0 (0%)	0 (0%)	0 (0%)	57 (67.1%)	0 (0%)	0 (0%)	0 (0%)	0 (0%)	80 (87.9%)	36 (48.6%)	0 (0%)	103 (37.7%)	30 (50.0%)	0 (0%)
65 (100%)	147 (100%)	82 (100%)	163 (100%)	0 (0%)	174 (100%)	498 (100%)	85 (100%)	90 (100%)	0 (0%)	0 (0%)	159 (100%)	0 (0%)	0 (0%)	70 (100%)
0 (0%)	24 (16.3%)	39 (47.6%)	39 (23.9%)	32 (37.6%)	9 (5.2%)	1 (0.2%)	2 (2.4%)	40 (44.4%)	17 (18.7%)	19 (25.7%)	14 (8.8%)	1 (0.4%)	6 (10.0%)	7 (10.0%)
8 (12.3%)	67 (45.6%)	27 (32.9%)	78 (47.9%)	34 (40.0%)	113 (64.9%)	12 (2.4%)	56 (65.9%)	30 (33.3%)	55 (60.4%)	37 (50.0%)	20 (12.6%)	27 (9.9%)	37 (61.7%)	25 (35.7%)
36 (55.4%)	27 (18.4%)	15 (18.3%)	36 (22.1%)	9 (10.6%)	42 (24.1%)	386 (77.5%)	22 (25.9%)	15 (16.7%)	14 (15.4%)	8 (10.8%)	92 (57.9%)	202 (74.0%)	11 (18.3%)	22 (31.4%)
19 (29.2%)	28 (19.0%)	0 (0%)	10 (6.1%)	10 (11.8%)	9 (5.2%)	80 (16.1%)	5 (5.9%)	5 (5.6%)	5 (5.5%)	10 (13.5%)	31 (19.5%)	43 (15.8%)	6 (10.0%)	13 (18.6%)
2 (3.1%)	1 (0.7%)	1 (1.2%)	0 (0%)	0 (0%)	1 (0.6%)	19 (3.8%)	0 (0%)	0 (0%)	0 (0%)	0 (0%)	2 (1.3%)	0 (0%)	0 (0%)	3 (4.3%)
54 (83.1%)	140 (95.2%)	80 (97.6%)	159 (97.5%)	80 (94.1%)	145 (83.3%)	444 (89.2%)	82 (96.5%)	80 (88.9%)	69 (75.8%)	71 (95.9%)	131 (82.4%)	251 (91.9%)	50 (83.3%)	43 (61.4%)
11 (16.9%)	7 (4.8%)	2 (2.4%)	4 (2.5%)	5 (5.9%)	28 (16.1%)	54 (10.8%)	3 (3.5%)	10 (11.1%)	22 (24.2%)	3 (4.1%)	28 (17.6%)	22 (8.1%)	10 (16.7%)	27 (38.6%)
0 (0%)	0 (0%)	0 (0%)	0 (0%)	0 (0%)	1 (0.6%)	0 (0%)	0 (0%)	0 (0%)	0 (0%)	0 (0%)	0 (0%)	0 (0%)	0 (0%)	0 (0%)
43 (66.2%)	119 (81.0%)	63 (76.8%)	121 (74.2%)	76 (89.4%)	112 (64.4%)	408 (81.9%)	56 (65.9%)	62 (68.9%)	51 (56.0%)	61 (82.4%)	91 (57.2%)	229 (83.9%)	48 (80.0%)	52 (74.3%)
22 (33.8%)	28 (19.0%)	19 (23.2%)	42 (25.8%)	9 (10.6%)	61 (35.1%)	90 (18.1%)	29 (34.1%)	28 (31.1%)	40 (44.0%)	13 (17.6%)	68 (42.8%)	44 (16.1%)	12 (20.0%)	18 (25.7%)
0 (0%)	0 (0%)	0 (0%)	0 (0%)	0 (0%)	1 (0.6%)	0 (0%)	0 (0%)	0 (0%)	0 (0%)	0 (0%)	0 (0%)	0 (0%)	0 (0%)	0 (0%)

Lesotho	Liberia	Libya	Malawi	Mongolia	Morocco	Myanmar	Nepal	Niue	Qatar	Republic of Moldova	Sao Tome and Principe	Seychelles	Sri Lanka	Sudan
(N=134)	(N=78)	(N=169)	(N=86)	(N=553)	(N=182)	(N=529)	(N=131)	(N=55)	(N=157)	(N=551)	(N=124)	(N=249)	(N=301)	(N=272)
53.6 (8.52)	49.1 (9.40)	53.2 (7.55)	56.8 (9.38)	54.4 (9.11)	56.7 (8.78)	51.6 (8.40)	53.5 (9.65)	54.8 (6.73)	52.7 (7.98)	58.1 (7.52)	51.9 (10.2)	52.8 (8.20)	56.9 (8.45)	53.8 (9.52)
55.0 [26.0, 64.0]	50.0 [29.0, 64.0]	54.0 [35.0, 64.0]	57.0 [29.0, 69.0]	56.0 [27.0, 69.0]	58.0 [33.0, 69.0]	53.0 [25.0, 64.0]	54.0 [29.0, 69.0]	55.0 [34.0, 68.0]	54.0 [27.0, 64.0]	60.0 [28.0, 69.0]	52.0 [27.0, 69.0]	54.0 [28.0, 64.0]	59.0 [30.0, 69.0]	55.0 [30.0, 69.0]
10 (7.5%)	15 (19.2%)	7 (4.1%)	5 (5.8%)	44 (8.0%)	8 (4.4%)	47 (8.9%)	10 (7.6%)	1 (1.8%)	10 (6.4%)	14 (2.5%)	15 (12.1%)	18 (7.2%)	12 (4.0%)	21 (7.7%)
50 (37.3%)	39 (50.0%)	83 (49.1%)	21 (24.4%)	202 (36.5%)	57 (31.3%)	259 (49.0%)	57 (43.5%)	24 (43.6%)	74 (47.1%)	140 (25.4%)	55 (44.4%)	107 (43.0%)	90 (29.9%)	110 (40.4%)
74 (55.2%)	24 (30.8%)	79 (46.7%)	60 (69.8%)	307 (55.5%)	117 (64.3%)	223 (42.2%)	64 (48.9%)	30 (54.5%)	73 (46.5%)	397 (72.1%)	54 (43.5%)	124 (49.8%)	199 (66.1%)	141 (51.8%)
11 (8.2%)	27 (34.6%)	73 (43.2%)	20 (23.3%)	231 (41.8%)	39 (21.4%)	121 (22.9%)	60 (45.8%)	24 (43.6%)	73 (46.5%)	140 (25.4%)	19 (15.3%)	109 (43.8%)	88 (29.2%)	69 (25.4%)
123 (91.8%)	51 (65.4%)	96 (56.8%)	66 (76.7%)	322 (58.2%)	143 (78.6%)	408 (77.1%)	71 (54.2%)	31 (56.4%)	84 (53.5%)	411 (74.6%)	105 (84.7%)	140 (56.2%)	213 (70.8%)	203 (74.6%)
162 (23.8)	166 (23.7)	172 (22.6)	153 (17.6)	152 (18.2)	160 (19.8)	154 (18.5)	152 (18.5)	153 (18.5)	150 (14.9)	163 (21.2)	158 (22.3)	153 (17.1)	156 (18.7)	158 (20.4)
156 [131, 245]	160 [133, 219]	167 [131, 234]	150 [131, 222]	147 [131, 254]	157 [131, 227]	150 [131, 252]	148 [131, 227]	151 [132, 223]	148 [131, 203]	159 [131, 254]	151 [131, 254]	150 [131, 223]	153 [131, 222]	154 [131, 238]
102 (11.2)	102 (12.1)	95.2 (9.08)	94.4 (11.7)	94.7 (10.6)	93.2 (9.51)	96.5 (10.4)	95.8 (9.82)	90.0 (8.27)	94.3 (8.76)	96.8 (10.2)	101 (13.7)	97.5 (10.2)	95.1 (9.60)	97.8 (10.6)
102 [80.5, 146]	101 [81.0, 138]	94.0 [80.5, 134]	91.0 [81.5, 135]	92.5 [80.5, 143]	91.8 [80.5, 131]	95.0 [80.5, 141]	94.5 [80.5, 131]	88.0 [80.5, 117]	92.5 [80.5, 124]	95.5 [80.5, 139]	99.0 [80.5, 140]	96.0 [80.5, 138]	93.5 [81.0, 140]	96.5 [80.5, 139]
0 (0%)	0 (0%)	0 (0%)	53 (61.6%)	197 (35.6%)	51 (28.0%)	0 (0%)	93 (71.0%)	0 (0%)	0 (0%)	211 (38.3%)	49 (39.5%)	0 (0%)	0 (0%)	116 (42.6%)
0 (0%)	0 (0%)	0 (0%)	33 (38.4%)	356 (64.4%)	131 (72.0%)	0 (0%)	38 (29.0%)	0 (0%)	0 (0%)	340 (61.7%)	75 (60.5%)	0 (0%)	0 (0%)	156 (57.4%)
134 (100%)	78 (100%)	169 (100%)	0 (0%)	0 (0%)	0 (0%)	529 (100%)	0 (0%)	55 (100%)	157 (100%)	0 (0%)	0 (0%)	249 (100%)	301 (100%)	0 (0%)
8 (6.0%)	37 (47.4%)	50 (29.6%)	24 (27.9%)	8 (1.4%)	117 (64.3%)	63 (11.9%)	60 (45.8%)	0 (0%)	31 (19.7%)	1 (0.2%)	19 (15.3%)	23 (9.2%)	19 (6.3%)	126 (46.3%)
91 (67.9%)	9 (11.5%)	49 (29.0%)	51 (59.3%)	49 (8.9%)	33 (18.1%)	318 (60.1%)	35 (26.7%)	7 (12.7%)	41 (26.1%)	6 (1.1%)	91 (73.4%)	195 (78.3%)	59 (19.6%)	58 (21.3%)
28 (20.9%)	24 (30.8%)	50 (29.6%)	7 (8.1%)	269 (48.6%)	25 (13.7%)	119 (22.5%)	30 (22.9%)	25 (45.5%)	48 (30.6%)	204 (37.0%)	12 (9.7%)	25 (10.0%)	209 (69.4%)	56 (20.6%)
7 (5.2%)	8 (10.3%)	20 (11.8%)	4 (4.7%)	227 (41.0%)	7 (3.8%)	27 (5.1%)	6 (4.6%)	23 (41.8%)	37 (23.6%)	340 (61.7%)	1 (0.8%)	6 (2.4%)	13 (4.3%)	29 (10.7%)
0 (0%)	0 (0%)	0 (0%)	0 (0%)	0 (0%)	0 (0%)	2 (0.4%)	0 (0%)	0 (0%)	0 (0%)	0 (0%)	1 (0.8%)	0 (0%)	1 (0.3%)	3 (1.1%)
129 (96.3%)	71 (91.0%)	140 (82.8%)	84 (97.7%)	419 (75.8%)	179 (98.4%)	458 (86.6%)	105 (80.2%)	49 (89.1%)	134 (85.4%)	510 (92.6%)	124 (100%)	212 (85.1%)	286 (95.0%)	261 (96.0%)
5 (3.7%)	7 (9.0%)	29 (17.2%)	2 (2.3%)	134 (24.2%)	3 (1.6%)	71 (13.4%)	26 (19.8%)	6 (10.9%)	23 (14.6%)	41 (7.4%)	0 (0%)	37 (14.9%)	15 (5.0%)	11 (4.0%)
0 (0%)	0 (0%)	0 (0%)	0 (0%)	0 (0%)	0 (0%)	0 (0%)	0 (0%)	0 (0%)	0 (0%)	0 (0%)	0 (0%)	0 (0%)	0 (0%)	0 (0%)
111 (82.8%)	70 (89.7%)	103 (60.9%)	76 (88.4%)	468 (84.6%)	122 (67.0%)	424 (80.2%)	107 (81.7%)	31 (56.4%)	71 (45.2%)	399 (72.4%)	101 (81.5%)	208 (83.5%)	184 (61.1%)	205 (75.4%)
23 (17.2%)	8 (10.3%)	66 (39.1%)	10 (11.6%)	85 (15.4%)	60 (33.0%)	105 (19.8%)	24 (18.3%)	24 (43.6%)	86 (54.8%)	152 (27.6%)	23 (18.5%)	41 (16.5%)	117 (38.9%)	67 (24.6%)
0 (0%)	0 (0%)	0 (0%)	0 (0%)	0 (0%)	0 (0%)	0 (0%)	0 (0%)	0 (0%)	0 (0%)	0 (0%)	0 (0%)	0 (0%)	0 (0%)	0 (0%)

Tajikistan	Timor Leste	Tokelau	Tonga	Turkmenistan	Tuvalu	Uganda	United Republic of Tanzania	Vanuatu	Vietnam	Zambia	Overall
(N=220)	(N=71)	(N=40)	(N=210)	(N=354)	(N=59)	(N=46)	(N=110)	(N=69)	(N=109)	(N=91)	(N=10658)
53.2 (9.92)	49.8 (10.1)	53.0 (7.08)	54.7 (8.72)	54.5 (8.70)	53.5 (8.52)	51.5 (9.73)	51.1 (9.68)	53.3 (7.39)	56.7 (8.48)	53.0 (10.8)	54.4 (9.24)
54.0 [28.0, 69.0]	50.0 [27.0, 69.0]	54.0 [34.0, 64.0]	56.0 [29.0, 69.0]	56.0 [26.0, 69.0]	56.0 [30.0, 69.0]	50.5 [30.0, 68.0]	52.0 [25.0, 64.0]	54.0 [33.0, 64.0]	58.0 [33.0, 69.0]	53.0 [26.0, 69.0]	56.0 [25.0, 69.0]
21 (9.5%)	10 (14.1%)	2 (5.0%)	9 (4.3%)	19 (5.4%)	6 (10.2%)	4 (8.7%)	12 (10.9%)	2 (2.9%)	4 (3.7%)	11 (12.1%)	776 (7.3%)
97 (44.1%)	38 (53.5%)	18 (45.0%)	91 (43.3%)	142 (40.1%)	21 (35.6%)	28 (60.9%)	49 (44.5%)	36 (52.2%)	37 (33.9%)	37 (40.7%)	4080 (38.3%)
102 (46.4%)	23 (32.4%)	20 (50.0%)	110 (52.4%)	193 (54.5%)	32 (54.2%)	14 (30.4%)	49 (44.5%)	31 (44.9%)	68 (62.4%)	43 (47.3%)	5802 (54.4%)
58 (26.4%)	25 (35.2%)	15 (37.5%)	61 (29.0%)	124 (35.0%)	19 (32.2%)	7 (15.2%)	22 (20.0%)	23 (33.3%)	53 (48.6%)	19 (20.9%)	3323 (31.2%)
162 (73.6%)	46 (64.8%)	25 (62.5%)	149 (71.0%)	230 (65.0%)	40 (67.8%)	39 (84.8%)	88 (80.0%)	46 (66.7%)	56 (51.4%)	72 (79.1%)	7335 (68.8%)
161 (21.3)	161 (20.1)	150 (16.0)	157 (17.7)	157 (19.8)	160 (21.1)	164 (23.5)	168 (26.5)	161 (20.6)	152 (16.4)	158 (23.0)	158 (20.4)
157 [131, 240]	161 [131, 226]	147 [131, 202]	153 [131, 212]	153 [131, 234]	154 [132, 221]	157 [132, 216]	164 [131, 233]	156 [132, 212]	150 [131, 210]	152 [131, 231]	153 [131, 259]
100 (10.9)	98.2 (9.02)	96.0 (9.52)	96.0 (10.3)	98.3 (10.6)	98.3 (11.1)	103 (12.2)	101 (13.1)	95.6 (9.93)	95.2 (9.82)	97.2 (12.7)	96.6 (10.6)
97.5 [81.0, 141]	95.5 [81.5, 119]	95.5 [81.0, 124]	94.5 [80.5, 132]	96.5 [80.5, 136]	97.5 [81.0, 128]	100 [80.5, 135]	98.3 [80.5, 133]	93.5 [82.5, 124]	94.5 [80.5, 125]	93.0 [80.5, 134]	95.0 [80.5, 150]
0 (0%)	0 (0%)	0 (0%)	0 (0%)	143 (40.4%)	0 (0%)	25 (54.3%)	0 (0%)	0 (0%)	41 (37.6%)	45 (49.5%)	2130 (20.0%)
0 (0%)	0 (0%)	0 (0%)	0 (0%)	211 (59.6%)	0 (0%)	21 (45.7%)	0 (0%)	0 (0%)	68 (62.4%)	46 (50.5%)	2875 (27.0%)
220 (100%)	71 (100%)	40 (100%)	210 (100%)	0 (0%)	59 (100%)	0 (0%)	110 (100%)	69 (100%)	0 (0%)	0 (0%)	5653 (53.0%)
3 (1.4%)	21 (29.6%)	0 (0%)	0 (0%)	1 (0.3%)	1 (1.7%)	13 (28.3%)	21 (19.1%)	3 (4.3%)	4 (3.7%)	15 (16.5%)	1743 (16.4%)
42 (19.1%)	19 (26.8%)	0 (0%)	19 (9.0%)	0 (0%)	38 (64.4%)	12 (26.1%)	70 (63.6%)	54 (78.3%)	36 (33.0%)	43 (47.3%)	2922 (27.4%)
136 (61.8%)	23 (32.4%)	22 (55.0%)	160 (76.2%)	283 (79.9%)	19 (32.2%)	15 (32.6%)	16 (14.5%)	10 (14.5%)	49 (45.0%)	20 (22.0%)	4146 (38.9%)
39 (17.7%)	7 (9.9%)	18 (45.0%)	31 (14.8%)	70 (19.8%)	1 (1.7%)	6 (13.0%)	3 (2.7%)	1 (1.4%)	20 (18.3%)	13 (14.3%)	1771 (16.6%)
0 (0%)	1 (1.4%)	0 (0%)	0 (0%)	0 (0%)	0 (0%)	0 (0%)	0 (0%)	1 (1.4%)	0 (0%)	0 (0%)	76 (0.7%)
211 (95.9%)	57 (80.3%)	25 (62.5%)	187 (89.0%)	341 (96.3%)	44 (74.6%)	44 (95.7%)	105 (95.5%)	64 (92.8%)	87 (79.8%)	86 (94.5%)	9486 (89.0%)
9 (4.1%)	14 (19.7%)	15 (37.5%)	23 (11.0%)	13 (3.7%)	15 (25.4%)	2 (4.3%)	5 (4.5%)	5 (7.2%)	22 (20.2%)	5 (5.5%)	1171 (11.0%)
0 (0%)	0 (0%)	0 (0%)	0 (0%)	0 (0%)	0 (0%)	0 (0%)	0 (0%)	0 (0%)	0 (0%)	0 (0%)	1 (0.0%)
192 (87.3%)	66 (93.0%)	18 (45.0%)	0 (0%)	320 (90.4%)	47 (79.7%)	42 (91.3%)	96 (87.3%)	45 (65.2%)	96 (88.1%)	76 (83.5%)	8140 (76.4%)
28 (12.7%)	5 (7.0%)	22 (55.0%)	0 (0%)	34 (9.6%)	12 (20.3%)	4 (8.7%)	14 (12.7%)	22 (31.9%)	13 (11.9%)	15 (16.5%)	2305 (21.6%)
0 (0%)	0 (0%)	0 (0%)	210 (100%)	0 (0%)	0 (0%)	0 (0%)	0 (0%)	2 (2.9%)	0 (0%)	0 (0%)	213 (2.0%)

Supplementary Table 2. Mean systolic blood pressure (mmHg) by world region, income group and country.

Region	Income	Data year	Country	Mean	95% lower CI	95% upper CI
Africa	Lower	2010	Eritrea	155.5	150.0	160.9
Africa	Lower	2011	Comoros	161.4	157.4	165.4
Africa	Lower	2011	Liberia	166.2	160.5	172.0
Africa	Lower	2012	United Republic of Tanzania	169.2	163.2	175.1
Africa	Lower	2014	Uganda	160.1	151.9	168.2
Africa	Lower	2015	Benin	170.4	165.8	175.0
Africa	Lower	2015	Ethiopia	162.1	153.3	171.0
Africa	Lower	2017	Malawi	152.8	147.8	157.8
Africa	Lower-Middle	2007	Cabo Verde	159.3	152.5	166.1
Africa	Lower-Middle	2012	Lesotho	157.8	152.9	162.7
Africa	Lower-Middle	2014	Eswatini	160.0	155.8	164.1
Africa	Lower-Middle	2015	Kenya	162.0	153.0	170.9
Africa	Lower-Middle	2017	Zambia	156.7	152.2	161.2
Africa	Lower-Middle	2019	Sao Tome and Principe	155.4	150.8	160.1
Africa	Upper-Middle	2004	Seychelles	151.9	149.8	154.0
Africa	Upper-Middle	2014	Botswana	155.1	150.5	159.8
Africa	Upper-Middle	2017	Algeria	158.9	156.6	161.2
Americas	Upper-Middle	2016	Guyana	156.0	139.8	172.2
Americas	Upper-Middle	2018	Ecuador	147.5	144.2	150.8
Eastern Mediterranean	High	2012	Qatar	149.5	146.9	152.2
Eastern Mediterranean	High	2014	Kuwait	146.6	143.8	149.4
Eastern Mediterranean	Lower	2018	Afghanistan	152.3	148.4	156.3
Eastern Mediterranean	Lower-Middle	2013	Kyrgyzstan	166.9	163.0	170.9
Eastern Mediterranean	Lower-Middle	2016	Sudan	158.2	155.3	161.1
Eastern Mediterranean	Lower-Middle	2017	Morocco	158.5	155.6	161.4

Eastern Mediterranean	Upper-Middle	2009	Libya	171.9	167.8	176.0
Eastern Mediterranean	Upper-Middle	2015	Iraq	154.8	151.7	157.9
Eastern Mediterranean	Upper-Middle	2017	Lebanon	150.4	145.8	154.9
Eastern Mediterranean	Upper-Middle	2019	Jordan	150.6	144.9	156.4
Europe	Lower	2017	Tajikistan	160.8	157.2	164.4
Europe	Lower-Middle	2013	Republic of Moldova	162.4	160.0	164.9
Europe	Lower-Middle	2016	Armenia	160.7	156.8	164.6
Europe	Lower-Middle	2016	Georgia	161.0	158.4	163.6
Europe	Upper-Middle	2017	Azerbaijan	157.9	155.3	160.5
Europe	Upper-Middle	2017	Belarus	159.4	157.9	160.8
Europe	Upper-Middle	2018	Turkmenistan	155.8	153.2	158.3
Southeast Asia	Lower-Middle	2014	Myanmar	154.1	151.4	156.9
Southeast Asia	Lower-Middle	2014	Timor-Leste	160.5	156.5	164.5
Southeast Asia	Lower-Middle	2015	Sri Lanka	156.5	154.0	159.0
Southeast Asia	Lower-Middle	2018	Bangladesh	154.4	151.5	157.3
Southeast Asia	Lower-Middle	2019	Bhutan	148.6	146.5	150.8
Southeast Asia	Lower-Middle	2019	Nepal	153.4	148.7	158.0
Western Pacific	High	2016	Brunei Darussalam	151.5	147.0	155.9
Western Pacific	Lower	2010	Cambodia	155.2	150.4	160.1
Western Pacific	Lower-Middle	2011	Fiji	162.7	159.8	165.6
Western Pacific	Lower-Middle	2011	Vanuatu	158.6	153.3	163.9
Western Pacific	Lower-Middle	2013	Lao People's Democratic Republic	162.5	154.7	170.4
Western Pacific	Lower-Middle	2015	Vietnam	151.8	148.3	155.2
Western Pacific	Lower-Middle	2019	Mongolia	152.0	150.1	154.0
Western Pacific	No data	2012	Niue	153.5	148.6	158.4
Western Pacific	No data	2014	Tokelau	150.6	143.4	157.9
Western Pacific	No data	2015	Cook Islands	150.2	148.1	152.2
Western Pacific	Upper-Middle	2004	American Samoa	155.7	152.9	158.5

Western Pacific	Upper-Middle	2015	Tuvalu	157.2	148.2	166.2
Western Pacific	Upper-Middle	2017	Tonga	156.7	153.9	159.4

CI: confidence interval.

Supplementary Table 3. Median systolic blood pressure (mmHg) by world region, income group and country.

Region	Income	Data year	Country	q25	q50 (median)	q75
Africa	Lower	2010	Eritrea	137.0	152.5	170.5
Africa	Lower	2011	Comoros	143.5	158.5	178.0
Africa	Lower	2011	Liberia	146.0	159.0	185.0
Africa	Lower	2012	United Republic of Tanzania	147.5	165.5	187.5
Africa	Lower	2014	Uganda	142.0	153.5	177.5
Africa	Lower	2015	Benin	153.0	171.5	181.0
Africa	Lower	2015	Ethiopia	143.5	153.0	175.5
Africa	Lower	2017	Malawi	140.5	152.0	158.0
Africa	Lower-Middle	2007	Cabo Verde	146.5	154.5	169.5
Africa	Lower-Middle	2012	Lesotho	140.5	153.5	171.0
Africa	Lower-Middle	2014	Eswatini	139.5	151.5	173.0
Africa	Lower-Middle	2015	Kenya	137.0	155.0	176.0
Africa	Lower-Middle	2017	Zambia	143.0	152.0	162.0
Africa	Lower-Middle	2019	Sao Tome and Principe	140.5	150.0	165.5
Africa	Upper-Middle	2004	Seychelles	139.5	149.0	160.5
Africa	Upper-Middle	2014	Botswana	139.5	147.5	164.0
Africa	Upper-Middle	2017	Algeria	144.0	156.0	171.0
Americas	Upper-Middle	2016	Guyana	138.5	147.0	159.5
Americas	Upper-Middle	2018	Ecuador	136.5	143.5	154.0
Eastern Mediterranean	High	2012	Qatar	140.5	147.5	155.5
Eastern Mediterranean	High	2014	Kuwait	140.0	142.5	150.0
Eastern Mediterranean	Lower	2018	Afghanistan	144.0	149.5	158.0
Eastern Mediterranean	Lower-Middle	2013	Kyrgyzstan	150.5	162.5	180.5
Eastern Mediterranean	Lower-Middle	2016	Sudan	143.5	155.0	168.0
Eastern Mediterranean	Lower-Middle	2017	Morocco	143.0	155.5	170.0

Eastern Mediterranean	Upper-Middle	2009	Libya	155.5	170.5	186.5
Eastern Mediterranean	Upper-Middle	2015	Iraq	148.0	152.5	160.0
Eastern Mediterranean	Upper-Middle	2017	Lebanon	136.0	145.0	164.5
Eastern Mediterranean	Upper-Middle	2019	Jordan	136.5	144.0	161.0
Europe	Lower	2017	Tajikistan	146.0	157.0	168.0
Europe	Lower-Middle	2013	Republic of Moldova	146.5	157.5	172.5
Europe	Lower-Middle	2016	Armenia	146.5	157.0	171.5
Europe	Lower-Middle	2016	Georgia	145.5	158.0	174.0
Europe	Upper-Middle	2017	Azerbaijan	145.0	154.0	169.0
Europe	Upper-Middle	2017	Belarus	144.5	155.0	169.0
Europe	Upper-Middle	2018	Turkmenistan	140.0	151.0	167.5
Southeast Asia	Lower-Middle	2014	Myanmar	138.0	149.5	167.0
Southeast Asia	Lower-Middle	2014	Timor-Leste	144.0	158.5	170.0
Southeast Asia	Lower-Middle	2015	Sri Lanka	142.0	153.0	168.0
Southeast Asia	Lower-Middle	2018	Bangladesh	140.0	151.5	165.0
Southeast Asia	Lower-Middle	2019	Bhutan	136.0	146.0	156.5
Southeast Asia	Lower-Middle	2019	Nepal	138.5	147.5	162.5
Western Pacific	High	2016	Brunei Darussalam	138.0	145.5	156.5
Western Pacific	Lower	2010	Cambodia	140.0	152.0	164.5
Western Pacific	Lower-Middle	2011	Fiji	146.5	158.0	174.0
Western Pacific	Lower-Middle	2011	Vanuatu	144.5	155.0	168.5
Western Pacific	Lower-Middle	2013	Lao People's Democratic Republic	147.0	158.0	178.0
Western Pacific	Lower-Middle	2015	Vietnam	138.0	150.0	161.0
Western Pacific	Lower-Middle	2019	Mongolia	138.0	147.5	160.5
Western Pacific	No data	2012	Niue	139.5	150.5	162.5
Western Pacific	No data	2014	Tokelau	137.5	146.5	161.0
Western Pacific	No data	2015	Cook Islands	142.0	146.5	157.0
Western Pacific	Upper-Middle	2004	American Samoa	142.5	150.5	165.0

Western Pacific	Upper-Middle	2015	Tuvalu	143.5	148.0	167.0
Western Pacific	Upper-Middle	2017	Tonga	143.5	152.0	167.0

q25 refers to the percentile 25th, q50 refers to the percentile 50th or the median; and q75 refers to the percentile 75th.

Supplementary Table 4. Mean systolic blood pressure (mmHg) by country and sex.

Country	Sex	Mean	95% lower CI	95% upper CI
Algeria	Men	160.9	156.8	164.9
Algeria	Women	157.8	154.9	160.6
Benin	Men	162.2	156.9	167.6
Benin	Women	175.4	169.6	181.2
Botswana	Men	160.3	149.9	170.8
Botswana	Women	151.9	148.6	155.2
Cabo Verde	Men	158.3	152.1	164.6
Cabo Verde	Women	159.8	152.1	167.5
Comoros	Men	159.2	151.6	166.8
Comoros	Women	162.5	158.2	166.7
Eritrea	Men	155.6	142.2	168.9
Eritrea	Women	155.4	149.2	161.6
Eswatini	Men	154.9	145.9	164.0
Eswatini	Women	161.8	156.0	167.7
Ethiopia	Men	158.5	147.1	169.9
Ethiopia	Women	167.6	155.3	179.8
Kenya	Men	161.2	143.7	178.8
Kenya	Women	162.3	151.8	172.8
Lesotho	Men	145.1	138.2	151.9
Lesotho	Women	160.3	155.1	165.5
Liberia	Men	164.2	155.7	172.8
Liberia	Women	167.4	162.1	172.8
Malawi	Men	150.1	144.9	155.4
Malawi	Women	153.8	147.5	160.0
Sao Tome and Principe	Men	153.4	143.5	163.2
Sao Tome and Principe	Women	155.6	150.7	160.6
Seychelles	Men	153.0	150.3	155.8
Seychelles	Women	150.8	147.7	153.9
Uganda	Men	146.4	138.3	154.4
Uganda	Women	162.8	153.4	172.1
United Republic of Tanzania	Men	174.8	161.3	188.4
United Republic of Tanzania	Women	166.7	160.6	172.9
Zambia	Men	161.8	151.8	171.8
Zambia	Women	154.4	149.9	158.9
Ecuador	Men	148.2	144.1	152.2

Ecuador	Women	146.9	141.4	152.5
Guyana	Men	164.4	136.0	192.7
Guyana	Women	147.1	143.6	150.7
Afghanistan	Men	153.9	147.8	160.0
Afghanistan	Women	151.5	147.7	155.3
Iraq	Men	156.8	151.7	161.9
Iraq	Women	153.7	149.7	157.7
Jordan	Men	150.7	142.1	159.3
Jordan	Women	150.5	144.2	156.8
Kuwait	Men	148.1	143.5	152.6
Kuwait	Women	145.1	141.8	148.5
Kyrgyzstan	Men	176.3	168.5	184.1
Kyrgyzstan	Women	163.1	159.3	166.8
Lebanon	Men	154.0	147.8	160.2
Lebanon	Women	145.4	141.2	149.7
Libya	Men	172.8	167.3	178.2
Libya	Women	171.4	166.0	176.8
Morocco	Men	156.2	150.4	161.9
Morocco	Women	159.4	156.1	162.6
Qatar	Men	149.5	146.1	153.0
Qatar	Women	149.5	144.1	154.9
Sudan	Men	155.7	150.4	161.1
Sudan	Women	159.3	156.1	162.6
Armenia	Men	152.9	147.2	158.6
Armenia	Women	165.0	160.7	169.2
Azerbaijan	Men	160.1	155.4	164.7
Azerbaijan	Women	156.5	153.7	159.3
Belarus	Men	159.8	157.5	162.2
Belarus	Women	159.1	157.2	160.9
Georgia	Men	163.2	158.3	168.0
Georgia	Women	159.5	156.9	162.1
Republic of Moldova	Men	160.7	156.6	164.7
Republic of Moldova	Women	163.5	160.8	166.2
Tajikistan	Men	162.2	155.0	169.3
Tajikistan	Women	159.9	156.3	163.4
Turkmenistan	Men	153.8	150.4	157.2
Turkmenistan	Women	157.2	153.8	160.7
Bangladesh	Men	154.7	149.4	160.1
Bangladesh	Women	154.2	151.0	157.4

Bhutan	Men	146.1	142.3	150.0
Bhutan	Women	151.1	148.3	153.9
Myanmar	Men	151.9	147.9	155.9
Myanmar	Women	155.4	150.1	160.6
Nepal	Men	153.5	146.3	160.8
Nepal	Women	153.2	147.4	159.0
Sri Lanka	Men	155.7	152.0	159.4
Sri Lanka	Women	157.0	153.7	160.2
Timor-Leste	Men	162.0	156.5	167.6
Timor-Leste	Women	159.5	153.7	165.3
American Samoa	Men	157.9	153.8	161.9
American Samoa	Women	153.1	150.1	156.2
Brunei Darussalam	Men	151.1	144.7	157.5
Brunei Darussalam	Women	151.8	145.9	157.8
Cambodia	Men	155.6	146.2	165.1
Cambodia	Women	155.1	149.5	160.6
Cook Islands	Men	147.2	144.0	150.4
Cook Islands	Women	154.2	148.4	159.9
Fiji	Men	159.7	155.5	163.9
Fiji	Women	164.8	161.0	168.6
Lao People's Democratic Republic	Men	171.6	155.6	187.6
Lao People's Democratic Republic	Women	158.6	151.1	166.1
Mongolia	Men	153.2	150.2	156.2
Mongolia	Women	151.0	148.6	153.3
Niue	Men	156.3	148.4	164.1
Niue	Women	151.1	145.0	157.1
Tokelau	Men	148.5	138.8	158.1
Tokelau	Women	151.8	143.4	160.2
Tonga	Men	159.9	154.3	165.5
Tonga	Women	155.3	152.9	157.8
Tuvalu	Men	153.7	147.7	159.6
Tuvalu	Women	160.3	150.7	169.9
Vanuatu	Men	159.7	152.5	166.9
Vanuatu	Women	158.2	151.7	164.7
Vietnam	Men	150.6	146.4	154.8
Vietnam	Women	153.0	147.5	158.5

CI: confidence interval.

Supplementary Table 5. Mean systolic blood pressure by country and age groups.

Country	Age group	Mean	95% lower CI	95% upper CI
Afghanistan	<40	152.0	145.3	158.7
Afghanistan	40-54	151.4	147.0	155.7
Afghanistan	55+	153.8	148.1	159.4
Algeria	<40	146.2	139.7	152.6
Algeria	40-54	157.7	154.6	160.7
Algeria	55+	161.6	158.2	164.9
American Samoa	<40	153.2	141.6	164.9
American Samoa	40-54	153.7	150.9	156.6
American Samoa	55+	160.4	156.6	164.1
Armenia	<40	164.7	152.0	177.3
Armenia	40-54	156.2	149.4	163.0
Armenia	55+	162.9	158.5	167.4
Azerbaijan	<40	161.7	146.7	176.6
Azerbaijan	40-54	152.5	148.5	156.5
Azerbaijan	55+	161.0	158.1	163.8
Bangladesh	<40	150.6	145.3	155.9
Bangladesh	40-54	152.0	148.3	155.7
Bangladesh	55+	159.9	154.6	165.2
Belarus	<40	150.9	145.9	155.9
Belarus	40-54	157.7	154.8	160.7
Belarus	55+	161.0	159.1	162.8
Benin	<40	179.0	167.8	190.3
Benin	40-54	170.3	161.8	178.8
Benin	55+	167.4	162.3	172.5
Bhutan	<40	141.0	135.6	146.3
Bhutan	40-54	147.8	144.3	151.3
Bhutan	55+	151.6	148.6	154.6
Botswana	<40	146.7	142.3	151.0
Botswana	40-54	154.4	145.4	163.5
Botswana	55+	160.4	154.0	166.8
Brunei Darussalam	<40	144.3	139.7	148.9
Brunei Darussalam	40-54	152.4	144.9	160.0
Brunei Darussalam	55+	152.3	146.6	158.0
Cabo Verde	<40	156.2	139.7	172.6
Cabo Verde	40-54	161.3	153.7	168.9
Cabo Verde	55+	158.3	146.6	170.0

Cambodia	<40	147.8	141.3	154.4
Cambodia	40-54	152.5	145.9	159.1
Cambodia	55+	163.7	154.4	173.0
Comoros	<40	150.8	142.8	158.7
Comoros	40-54	164.0	157.7	170.4
Comoros	55+	163.8	157.9	169.6
Cook Islands	<40	141.9	139.7	144.0
Cook Islands	40-54	150.2	147.5	152.9
Cook Islands	55+	151.4	148.8	154.0
Ecuador	<40	138.2	135.7	140.6
Ecuador	40-54	146.4	140.2	152.6
Ecuador	55+	149.4	145.6	153.1
Eritrea	<40	159.5	139.2	179.8
Eritrea	40-54	154.5	145.8	163.3
Eritrea	55+	155.9	149.2	162.5
Eswatini	<40	148.9	143.4	154.4
Eswatini	40-54	158.0	149.7	166.3
Eswatini	55+	162.1	156.9	167.3
Ethiopia	<40	162.4	135.1	189.7
Ethiopia	40-54	157.5	147.9	167.1
Ethiopia	55+	164.8	153.0	176.6
Fiji	<40	154.2	143.8	164.6
Fiji	40-54	159.8	155.3	164.4
Fiji	55+	166.6	162.2	171.0
Georgia	<40	147.5	143.9	151.2
Georgia	40-54	158.6	154.0	163.2
Georgia	55+	163.2	160.1	166.2
Guyana	<40	146.1	137.5	154.7
Guyana	40-54	145.7	139.4	152.0
Guyana	55+	163.0	138.2	187.7
Iraq	<40	152.3	146.0	158.5
Iraq	40-54	155.5	151.6	159.4
Iraq	55+	154.9	150.0	159.7
Jordan	<40	142.2	137.9	146.5
Jordan	40-54	148.7	136.3	161.2
Jordan	55+	152.6	145.6	159.6
Kenya	<40	137.9	131.2	144.5
Kenya	40-54	162.6	144.9	180.2
Kenya	55+	165.0	156.3	173.7

Kuwait	<40	144.9	139.0	150.7
Kuwait	40-54	146.1	142.5	149.7
Kuwait	55+	147.5	142.4	152.7
Kyrgyzstan	<40	143.6	141.7	145.6
Kyrgyzstan	40-54	163.5	157.8	169.3
Kyrgyzstan	55+	170.0	165.5	174.5
Lao People's Democratic Republic	<40	156.9	144.8	169.0
Lao People's Democratic Republic	40-54	158.6	149.0	168.3
Lao People's Democratic Republic	55+	167.7	154.4	181.0
Lebanon	<40	137.7	133.5	141.9
Lebanon	40-54	151.8	143.8	159.9
Lebanon	55+	150.5	144.0	156.9
Lesotho	<40	135.0	130.1	139.8
Lesotho	40-54	157.5	150.7	164.3
Lesotho	55+	165.7	159.4	171.9
Liberia	<40	160.1	148.1	172.0
Liberia	40-54	170.7	162.0	179.4
Liberia	55+	162.1	151.8	172.5
Libya	<40	164.2	154.6	173.8
Libya	40-54	171.3	164.5	178.2
Libya	55+	173.0	166.7	179.3
Malawi	<40	144.4	137.8	151.0
Malawi	40-54	147.4	141.0	153.7
Malawi	55+	157.2	151.1	163.3
Mongolia	<40	147.9	144.5	151.3
Mongolia	40-54	150.5	147.7	153.3
Mongolia	55+	154.5	151.6	157.4
Morocco	<40	150.9	144.1	157.8
Morocco	40-54	155.1	150.5	159.6
Morocco	55+	160.8	157.0	164.6
Myanmar	<40	150.2	139.9	160.5
Myanmar	40-54	152.8	150.1	155.6
Myanmar	55+	156.2	153.6	158.9
Nepal	<40	143.9	137.8	150.0
Nepal	40-54	150.3	143.6	156.9
Nepal	55+	158.1	150.7	165.6
Niue	<40	134.5	134.5	134.5
Niue	40-54	152.0	144.8	159.3
Niue	55+	155.5	148.7	162.2

Qatar	<40	144.6	139.0	150.1
Qatar	40-54	146.3	143.1	149.5
Qatar	55+	153.6	149.8	157.4
Republic of Moldova	<40	155.7	149.7	161.7
Republic of Moldova	40-54	158.0	153.9	162.1
Republic of Moldova	55+	165.8	163.0	168.7
Sao Tome and Principe	<40	141.9	138.6	145.2
Sao Tome and Principe	40-54	156.8	149.6	164.1
Sao Tome and Principe	55+	160.3	154.5	166.0
Seychelles	<40	148.6	143.3	154.0
Seychelles	40-54	149.7	146.7	152.8
Seychelles	55+	155.6	152.5	158.7
Sri Lanka	<40	145.8	140.2	151.5
Sri Lanka	40-54	150.7	147.0	154.4
Sri Lanka	55+	160.3	157.2	163.4
Sudan	<40	162.7	153.8	171.6
Sudan	40-54	155.1	150.6	159.5
Sudan	55+	160.6	156.5	164.7
Tajikistan	<40	152.7	146.7	158.6
Tajikistan	40-54	160.0	154.5	165.5
Tajikistan	55+	164.4	159.1	169.7
Timor-Leste	<40	153.1	146.8	159.4
Timor-Leste	40-54	158.0	152.2	163.9
Timor-Leste	55+	168.8	158.8	178.7
Tokelau	<40	149.6	138.1	161.0
Tokelau	40-54	151.7	135.7	167.7
Tokelau	55+	149.5	147.4	151.6
Tonga	<40	154.8	149.6	159.9
Tonga	40-54	158.6	153.6	163.5
Tonga	55+	155.2	151.0	159.4
Turkmenistan	<40	140.8	135.8	145.8
Turkmenistan	40-54	154.9	150.8	159.0
Turkmenistan	55+	158.4	155.1	161.8
Tuvalu	<40	148.5	146.6	150.5
Tuvalu	40-54	161.6	151.1	172.1
Tuvalu	55+	157.4	147.5	167.2
Uganda	<40	150.7	142.7	158.8
Uganda	40-54	159.0	147.8	170.2
Uganda	55+	164.6	150.3	178.8

United Republic of Tanzania	<40	155.0	140.3	169.7
United Republic of Tanzania	40-54	167.2	159.5	175.0
United Republic of Tanzania	55+	176.0	167.1	184.9
Vanuatu	<40	146.1	140.6	151.6
Vanuatu	40-54	152.7	146.5	158.9
Vanuatu	55+	169.1	161.3	177.0
Vietnam	<40	168.8	158.0	179.5
Vietnam	40-54	151.8	145.6	158.0
Vietnam	55+	150.9	146.7	155.0
Zambia	<40	145.5	141.0	150.0
Zambia	40-54	156.2	149.1	163.3
Zambia	55+	163.0	155.2	170.8

CI: confidence interval.

Supplementary Table 6. Mean systolic blood pressure (mmHg) by country and level of education.

Country	Education	Mean	95% lower CI	95% upper CI
Afghanistan	None	152.3	147.9	156.8
Afghanistan	Some Primary/Primary	149.9	144.7	155.1
Afghanistan	Secondary/High	158.3	151.0	165.6
Afghanistan	University+	152.1	143.3	160.9
Algeria	None	162.9	158.9	166.9
Algeria	Some Primary/Primary	159.7	155.7	163.8
Algeria	Secondary/High	152.7	148.3	157.1
Algeria	University+	151.5	144.1	158.9
American Samoa	Some Primary/Primary	166.1	130.1	202.1
American Samoa	Secondary/High	156.1	152.4	159.9
American Samoa	University+	152.2	146.1	158.2
Armenia	None	156.5	156.5	156.5
Armenia	Some Primary/Primary	165.3	162.4	168.2
Armenia	Secondary/High	162.3	157.7	166.9
Armenia	University+	154.3	148.1	160.5
Azerbaijan	None	171.0	146.5	195.6
Azerbaijan	Some Primary/Primary	165.3	151.3	179.2
Azerbaijan	Secondary/High	159.5	155.1	163.9
Azerbaijan	University+	155.8	152.8	158.9
Bangladesh	None	155.8	151.8	159.7
Bangladesh	Some Primary/Primary	153.2	147.8	158.6
Bangladesh	Secondary/High	155.5	149.9	161.1
Bangladesh	University+	151.9	144.2	159.6
Belarus	None	192.1	140.1	244.1
Belarus	Some Primary/Primary	163.0	160.4	165.7

Belarus	Secondary/High	158.9	157.1	160.7
Belarus	University+	155.7	152.9	158.6
Benin	None	175.3	168.8	181.9
Benin	Some Primary/Primary	165.8	159.3	172.3
Benin	Secondary/High	167.4	155.5	179.4
Benin	University+	156.4	148.8	164.0
Bhutan	None	151.3	148.6	154.1
Bhutan	Some Primary/Primary	147.7	142.4	153.1
Bhutan	Secondary/High	145.3	140.7	150.0
Bhutan	University+	141.1	136.8	145.3
Botswana	None	163.6	155.4	171.8
Botswana	Some Primary/Primary	155.2	144.6	165.8
Botswana	Secondary/High	152.2	147.8	156.7
Botswana	University+	148.2	137.9	158.5
Brunei Darussalam	None	144.8	138.1	151.5
Brunei Darussalam	Some Primary/Primary	153.1	142.9	163.2
Brunei Darussalam	Secondary/High	150.6	144.2	157.0
Brunei Darussalam	University+	152.6	143.2	162.1
Cabo Verde	None	163.6	156.1	171.2
Cabo Verde	Some Primary/Primary	159.4	144.6	174.3
Cabo Verde	Secondary/High	158.3	148.1	168.6
Cabo Verde	University+	146.2	143.5	148.9
Cambodia	None	158.3	151.8	164.9
Cambodia	Some Primary/Primary	156.5	148.9	164.1
Cambodia	Secondary/High	143.5	136.4	150.7
Comoros	None	165.6	160.6	170.5
Comoros	Some Primary/Primary	158.2	149.7	166.6
Comoros	Secondary/High	155.6	146.7	164.5

Comoros	University+	147.0	141.8	152.1
Cook Islands	Some Primary/Primary	153.1	148.7	157.5
Cook Islands	Secondary/High	147.6	144.7	150.6
Cook Islands	University+	153.4	151.7	155.2
Ecuador	None	155.2	147.9	162.6
Ecuador	Some Primary/Primary	146.9	141.3	152.4
Ecuador	Secondary/High	147.0	138.3	155.6
Ecuador	University+	144.4	141.6	147.3
Eritrea	None	158.7	151.3	166.1
Eritrea	Some Primary/Primary	151.4	143.4	159.3
Eritrea	Secondary/High	150.7	134.0	167.4
Eswatini	None	161.9	154.6	169.3
Eswatini	Some Primary/Primary	163.8	157.1	170.4
Eswatini	Secondary/High	149.9	142.9	156.9
Eswatini	University+	163.0	150.1	175.9
Ethiopia	None	161.5	148.4	174.6
Ethiopia	Some Primary/Primary	165.4	153.3	177.5
Ethiopia	Secondary/High	175.8	153.9	197.6
Ethiopia	University+	141.1	130.7	151.5
Fiji	None	166.6	156.8	176.4
Fiji	Some Primary/Primary	163.5	159.5	167.5
Fiji	Secondary/High	158.0	152.7	163.3
Fiji	University+	169.0	156.0	181.9
Georgia	None	160.5	160.5	160.5
Georgia	Some Primary/Primary	156.2	146.0	166.3
Georgia	Secondary/High	161.9	158.7	165.0
Georgia	University+	156.1	151.6	160.5
Guyana	None	137.5	129.9	145.1

Guyana	Some Primary/Primary	160.6	139.2	182.1
Guyana	Secondary/High	144.1	139.0	149.3
Guyana	University+	150.5	138.6	162.4
Iraq	None	155.6	150.2	161.0
Iraq	Some Primary/Primary	152.9	150.2	155.7
Iraq	Secondary/High	154.6	148.5	160.6
Iraq	University+	161.2	147.0	175.5
Jordan	None	163.3	148.3	178.3
Jordan	Some Primary/Primary	149.4	141.7	157.2
Jordan	Secondary/High	156.2	144.3	168.2
Jordan	University+	139.6	133.0	146.3
Kenya	None	162.6	145.8	179.4
Kenya	Some Primary/Primary	162.9	150.0	175.7
Kenya	Secondary/High	154.7	146.2	163.3
Kenya	University+	164.4	140.4	188.4
Kuwait	None	143.6	138.5	148.6
Kuwait	Some Primary/Primary	144.0	139.4	148.5
Kuwait	Secondary/High	146.8	142.5	151.2
Kuwait	University+	149.1	143.9	154.3
Kyrgyzstan	None	150.0	150.0	150.0
Kyrgyzstan	Some Primary/Primary	168.4	160.9	176.0
Kyrgyzstan	Secondary/High	168.3	163.7	172.8
Kyrgyzstan	University+	160.7	154.4	167.1
Lao People's Democratic Republic	None	167.7	155.3	180.0
Lao People's Democratic Republic	Some Primary/Primary	160.7	150.0	171.5
Lao People's Democratic Republic	Secondary/High	164.0	152.5	175.5
Lao People's Democratic Republic	University+	168.6	153.0	184.2
Lebanon	None	164.0	156.2	171.8

Lebanon	Some Primary/Primary	149.3	144.1	154.4
Lebanon	Secondary/High	149.8	143.5	156.1
Lebanon	University+	148.5	137.1	159.8
Lesotho	None	152.8	140.5	165.1
Lesotho	Some Primary/Primary	161.4	155.0	167.9
Lesotho	Secondary/High	156.2	149.0	163.5
Lesotho	University+	143.5	135.5	151.5
Liberia	None	169.6	163.0	176.1
Liberia	Some Primary/Primary	164.0	155.7	172.3
Liberia	Secondary/High	164.4	152.7	176.2
Liberia	University+	159.7	139.4	180.0
Libya	None	171.1	165.3	176.9
Libya	Some Primary/Primary	178.5	170.2	186.7
Libya	Secondary/High	166.4	160.3	172.5
Libya	University+	174.6	155.0	194.2
Malawi	None	162.9	151.8	173.9
Malawi	Some Primary/Primary	148.7	144.5	152.9
Malawi	Secondary/High	149.9	142.5	157.3
Malawi	University+	149.7	139.7	159.8
Mongolia	None	160.1	149.6	170.6
Mongolia	Some Primary/Primary	156.3	150.0	162.6
Mongolia	Secondary/High	153.5	150.4	156.6
Mongolia	University+	149.4	146.9	151.9
Morocco	None	160.8	157.2	164.4
Morocco	Some Primary/Primary	157.6	150.7	164.6
Morocco	Secondary/High	151.1	145.5	156.7
Morocco	University+	154.7	137.5	172.0
Myanmar	None	150.0	146.6	153.4

Myanmar	Some Primary/Primary	157.5	152.3	162.6
Myanmar	Secondary/High	150.3	145.7	154.8
Myanmar	University+	149.7	145.0	154.5
Nepal	None	156.2	148.8	163.7
Nepal	Some Primary/Primary	152.7	145.2	160.2
Nepal	Secondary/High	148.7	139.9	157.4
Nepal	University+	146.5	136.6	156.4
Niue	Some Primary/Primary	153.7	143.9	163.5
Niue	Secondary/High	157.2	148.0	166.4
Niue	University+	149.4	144.6	154.2
Qatar	None	151.3	144.1	158.6
Qatar	Some Primary/Primary	149.5	144.9	154.1
Qatar	Secondary/High	150.4	145.1	155.6
Qatar	University+	146.8	142.5	151.1
Republic of Moldova	None	132.5	132.5	132.5
Republic of Moldova	Some Primary/Primary	173.6	151.7	195.4
Republic of Moldova	Secondary/High	167.1	162.7	171.4
Republic of Moldova	University+	159.6	157.1	162.1
Sao Tome and Principe	None	164.1	155.4	172.8
Sao Tome and Principe	Some Primary/Primary	153.9	148.2	159.5
Sao Tome and Principe	Secondary/High	155.6	145.0	166.2
Sao Tome and Principe	University+	135.0	135.0	135.0
Seychelles	None	161.8	153.3	170.4
Seychelles	Some Primary/Primary	151.3	148.9	153.7
Seychelles	Secondary/High	150.8	145.8	155.8
Seychelles	University+	146.3	141.1	151.5
Sri Lanka	None	152.2	144.2	160.1
Sri Lanka	Some Primary/Primary	162.1	156.4	167.8

Sri Lanka	Secondary/High	155.4	152.6	158.2
Sri Lanka	University+	157.9	144.5	171.2
Sudan	None	162.5	157.8	167.2
Sudan	Some Primary/Primary	157.5	151.6	163.3
Sudan	Secondary/High	154.0	148.5	159.5
Sudan	University+	150.4	145.9	154.9
Tajikistan	None	151.1	140.6	161.5
Tajikistan	Some Primary/Primary	162.1	155.4	168.9
Tajikistan	Secondary/High	161.6	157.0	166.3
Tajikistan	University+	158.8	149.1	168.5
Timor-Leste	None	162.9	152.6	173.1
Timor-Leste	Some Primary/Primary	162.0	154.8	169.1
Timor-Leste	Secondary/High	159.1	151.7	166.4
Timor-Leste	University+	155.8	142.4	169.1
Tokelau	Secondary/High	150.8	141.1	160.5
Tokelau	University+	150.4	142.9	157.9
Tonga	Some Primary/Primary	159.6	148.4	170.7
Tonga	Secondary/High	156.3	153.5	159.2
Tonga	University+	156.8	150.8	162.8
Turkmenistan	None	136.5	136.5	136.5
Turkmenistan	Secondary/High	157.4	154.5	160.2
Turkmenistan	University+	150.5	146.5	154.5
Tuvalu	None	195.5	195.5	195.5
Tuvalu	Some Primary/Primary	161.8	154.0	169.6
Tuvalu	Secondary/High	150.2	144.2	156.1
Tuvalu	University+	152.0	152.0	152.0
Uganda	None	173.8	158.6	189.1
Uganda	Some Primary/Primary	159.1	145.5	172.6

Uganda	Secondary/High	153.8	142.8	164.8
Uganda	University+	148.8	135.5	162.1
United Republic of Tanzania	None	166.1	148.5	183.8
United Republic of Tanzania	Some Primary/Primary	167.9	161.2	174.6
United Republic of Tanzania	Secondary/High	179.5	168.7	190.3
United Republic of Tanzania	University+	156.6	121.6	191.5
Vanuatu	None	160.8	152.4	169.2
Vanuatu	Some Primary/Primary	160.2	154.6	165.8
Vanuatu	Secondary/High	154.8	142.0	167.6
Vanuatu	University+	146.5	146.5	146.5
Vietnam	None	161.7	146.6	176.9
Vietnam	Some Primary/Primary	152.7	146.2	159.2
Vietnam	Secondary/High	149.3	144.9	153.7
Vietnam	University+	156.2	146.2	166.1
Zambia	None	157.4	148.6	166.1
Zambia	Some Primary/Primary	156.6	149.3	164.0
Zambia	Secondary/High	158.1	147.2	169.1
Zambia	University+	154.1	146.1	162.2

CI: confidence interval.

Supplementary Table 7. Mean systolic blood pressure (mmHg) by country and urban/rural location.

Country	Location	Mean	95% lower CI	95% upper CI
Afghanistan	Rural	153.8	148.6	159.0
Afghanistan	Urban	151.6	146.1	157.0
Algeria	Rural	166.4	161.7	171.1
Algeria	Urban	156.7	154.1	159.2
Armenia	Rural	159.9	152.8	167.0
Armenia	Urban	161.1	156.5	165.7
Azerbaijan	Rural	160.2	156.7	163.7
Azerbaijan	Urban	155.9	152.1	159.7
Bangladesh	Rural	154.1	149.8	158.3
Bangladesh	Urban	154.9	151.4	158.4
Benin	Rural	177.7	169.9	185.5
Benin	Urban	167.0	161.7	172.3
Bhutan	Rural	151.3	148.1	154.6
Bhutan	Urban	145.6	143.6	147.7
Cambodia	Rural	157.1	151.2	163.0
Cambodia	Urban	152.6	144.6	160.6
Comoros	Rural	161.4	156.2	166.5
Comoros	Urban	161.5	155.1	167.9
Ethiopia	Rural	164.0	150.4	177.6
Ethiopia	Urban	159.2	150.6	167.8
Jordan	Rural	146.4	134.4	158.4
Jordan	Urban	151.0	144.8	157.1
Kenya	Rural	167.0	154.1	180.0
Kenya	Urban	158.7	146.8	170.6
Kyrgyzstan	Rural	165.9	161.8	170.1
Kyrgyzstan	Urban	169.4	160.8	177.9
Lao People's Democratic Republic	Rural	159.8	147.1	172.5
Lao People's Democratic Republic	Urban	165.4	156.5	174.3
Malawi	Rural	156.8	151.3	162.4
Malawi	Urban	144.8	139.1	150.6
Mongolia	Rural	151.8	148.4	155.3
Mongolia	Urban	152.2	149.9	154.5
Morocco	Rural	160.0	155.1	164.9
Morocco	Urban	158.0	154.6	161.5
Nepal	Rural	153.4	148.2	158.5
Nepal	Urban	153.4	144.0	162.8

Republic of Moldova	Rural	164.3	160.2	168.4
Republic of Moldova	Urban	160.4	158.0	162.9
Sao Tome and Principe	Rural	155.8	149.4	162.2
Sao Tome and Principe	Urban	155.3	149.1	161.5
Sudan	Rural	161.6	156.8	166.5
Sudan	Urban	156.3	152.8	159.7
Turkmenistan	Rural	162.3	158.4	166.2
Turkmenistan	Urban	151.6	148.7	154.4
Uganda	Rural	162.9	154.1	171.6
Uganda	Urban	155.4	139.9	171.0
Vietnam	Rural	155.9	150.4	161.4
Vietnam	Urban	147.4	143.8	151.0
Zambia	Rural	155.9	150.4	161.3
Zambia	Urban	157.2	150.9	163.5

CI: confidence interval.

Supplementary Table 8. Mean systolic blood pressure (mmHg) by country and current smoker status.

Country	Current smoker	Mean	95% lower CI	95% upper CI
Afghanistan	No	152.6	148.5	156.7
Afghanistan	Yes	145.7	138.9	152.6
Algeria	No	158.9	156.5	161.3
Algeria	Yes	158.6	147.8	169.4
American Samoa	No	157.5	155.0	159.9
American Samoa	Yes	150.5	143.6	157.3
Armenia	No	162.3	158.0	166.6
Armenia	Yes	152.3	145.9	158.7
Azerbaijan	No	158.6	155.7	161.5
Azerbaijan	Yes	153.9	149.7	158.1
Bangladesh	No	154.9	151.6	158.1
Bangladesh	Yes	151.5	145.8	157.2
Belarus	No	158.8	157.2	160.4
Belarus	Yes	162.0	158.2	165.7
Benin	No	170.5	165.8	175.2
Benin	Yes	168.9	149.2	188.6
Bhutan	No	148.4	146.0	150.9
Bhutan	Yes	155.1	134.8	175.4
Botswana	No	152.3	149.3	155.4
Botswana	Yes	168.3	149.3	187.2
Brunei Darussalam	No	151.8	147.0	156.7
Brunei Darussalam	Yes	148.2	139.4	157.1
Cabo Verde	No	157.2	151.3	163.1
Cabo Verde	Yes	178.7	178.7	178.7
Cambodia	No	153.5	148.3	158.6
Cambodia	Yes	163.1	149.6	176.6
Comoros	No	162.1	158.3	166.0
Comoros	Yes	156.4	142.4	170.3
Cook Islands	No	150.5	148.3	152.7
Cook Islands	Yes	148.3	145.4	151.2
Ecuador	No	147.7	144.3	151.2
Ecuador	Yes	142.2	134.2	150.3
Eritrea	No	155.2	149.9	160.5
Eritrea	Yes	167.0	120.7	213.3
Eswatini	No	159.7	155.5	163.9
Eswatini	Yes	170.4	149.8	190.9

Ethiopia	No	162.0	152.5	171.4
Ethiopia	Yes	164.3	150.7	177.9
Fiji	No	162.9	159.8	166.1
Fiji	Yes	161.8	154.6	169.0
Georgia	No	160.1	157.7	162.6
Georgia	Yes	164.0	156.5	171.6
Guyana	No	146.8	143.4	150.1
Guyana	Yes	212.7	179.5	245.9
Iraq	No	154.9	151.5	158.3
Iraq	Yes	154.3	147.7	160.8
Jordan	No	149.9	143.4	156.5
Jordan	Yes	153.4	138.8	168.1
Kenya	No	162.5	153.2	171.7
Kenya	Yes	156.1	130.7	181.4
Kuwait	No	147.1	143.7	150.4
Kuwait	Yes	144.2	141.1	147.4
Kyrgyzstan	No	165.6	161.9	169.3
Kyrgyzstan	Yes	176.9	163.2	190.7
Lao People's Democratic Republic	No	158.9	151.9	165.8
Lao People's Democratic Republic	Yes	178.0	155.8	200.1
Lebanon	No	150.6	144.6	156.6
Lebanon	Yes	149.9	144.7	155.1
Lesotho	No	158.6	153.7	163.4
Lesotho	Yes	145.0	126.0	164.1
Liberia	No	167.8	161.6	173.9
Liberia	Yes	151.1	145.0	157.2
Libya	No	171.9	167.6	176.2
Libya	Yes	172.1	161.9	182.3
Malawi	No	151.9	147.7	156.0
Malawi	Yes	160.7	133.1	188.2
Mongolia	No	151.6	149.5	153.7
Mongolia	Yes	153.3	149.3	157.2
Morocco	No	158.9	155.9	161.8
Morocco	Yes	146.6	134.5	158.7
Myanmar	No	154.0	151.1	157.0
Myanmar	Yes	154.8	148.8	160.7
Nepal	No	151.9	147.4	156.4
Nepal	Yes	160.3	145.8	174.8
Niue	No	152.7	147.9	157.5

Niue	Yes	160.1	138.1	182.1
Qatar	No	149.8	146.8	152.7
Qatar	Yes	148.5	141.3	155.8
Republic of Moldova	No	162.4	159.8	165.1
Republic of Moldova	Yes	162.7	157.5	167.9
Seychelles	No	152.2	149.9	154.5
Seychelles	Yes	150.6	146.2	155.1
Sri Lanka	No	156.8	154.2	159.4
Sri Lanka	Yes	150.7	141.9	159.4
Sudan	No	158.5	155.4	161.5
Sudan	Yes	152.9	145.8	160.0
Tajikistan	No	161.0	157.3	164.7
Tajikistan	Yes	155.7	148.0	163.5
Timor-Leste	No	161.5	156.7	166.2
Timor-Leste	Yes	157.4	149.7	165.2
Tokelau	No	148.6	143.8	153.3
Tokelau	Yes	153.7	142.1	165.3
Tonga	No	156.1	153.1	159.2
Tonga	Yes	161.0	152.3	169.7
Turkmenistan	No	155.9	153.3	158.5
Turkmenistan	Yes	154.2	145.1	163.3
Tuvalu	No	154.8	144.3	165.4
Tuvalu	Yes	163.8	155.4	172.1
Uganda	No	159.5	151.4	167.6
Uganda	Yes	180.6	148.0	213.2
United Republic of Tanzania	No	169.5	163.4	175.6
United Republic of Tanzania	Yes	163.2	146.9	179.4
Vanuatu	No	158.1	153.1	163.1
Vanuatu	Yes	165.4	141.3	189.4
Vietnam	No	153.2	149.2	157.3
Vietnam	Yes	144.8	140.0	149.6
Zambia	No	155.8	151.2	160.4
Zambia	Yes	179.9	156.6	203.1

CI: confidence interval.

Supplementary Table 9. Mean systolic blood pressure (mmHg) by country and self-reported diabetes status.

Country	Self-reported diabetes	Mean	95% lower CI	95% upper CI
Afghanistan	No	152.7	148.4	157.0
Afghanistan	Yes	151.3	144.5	158.2
Algeria	No	158.2	155.6	160.7
Algeria	Yes	160.5	155.9	165.0
American Samoa	No	155.5	152.2	158.7
American Samoa	Yes	155.9	151.0	160.9
Armenia	No	160.3	155.8	164.8
Armenia	Yes	162.9	155.0	170.8
Azerbaijan	No	157.6	154.7	160.6
Azerbaijan	Yes	159.0	153.6	164.4
Bangladesh	No	154.4	151.7	157.1
Bangladesh	Yes	154.3	146.9	161.7
Belarus	No	158.9	157.3	160.5
Belarus	Yes	161.6	157.9	165.3
Benin	No	171.6	166.5	176.7
Benin	Yes	161.1	151.4	170.9
Bhutan	No	148.2	145.4	150.9
Bhutan	Yes	151.2	145.5	156.8
Botswana	No	154.7	149.5	159.8
Botswana	Yes	158.5	150.6	166.4
Brunei Darussalam	No	150.4	144.9	156.0
Brunei Darussalam	Yes	153.8	147.0	160.7
Cabo Verde	No	158.1	149.6	166.6
Cabo Verde	Yes	178.7	158.8	198.6
Cambodia	No	156.3	151.3	161.4
Cambodia	Yes	151.1	139.9	162.2
Comoros	No	161.1	156.9	165.2
Comoros	Yes	163.9	155.3	172.5
Cook Islands	No	147.4	145.2	149.6
Cook Islands	Yes	155.6	152.8	158.4
Ecuador	No	146.7	143.0	150.4
Ecuador	Yes	150.6	143.5	157.8
Eritrea	No	154.5	148.6	160.5
Eritrea	Yes	158.3	147.0	169.7
Eswatini	No	160.2	155.1	165.4
Eswatini	Yes	159.4	151.3	167.4

Ethiopia	No	162.4	153.0	171.9
Ethiopia	Yes	157.0	145.3	168.7
Fiji	No	161.6	158.2	165.0
Fiji	Yes	164.1	158.4	169.8
Georgia	No	160.4	157.5	163.2
Georgia	Yes	164.3	159.8	168.8
Guyana	No	160.6	138.9	182.3
Guyana	Yes	145.1	141.8	148.3
Iraq	No	153.6	150.1	157.1
Iraq	Yes	157.8	152.6	163.1
Jordan	No	147.2	141.2	153.1
Jordan	Yes	154.3	143.3	165.3
Kenya	No	159.0	149.5	168.6
Kenya	Yes	180.4	164.6	196.3
Kuwait	No	147.2	142.8	151.6
Kuwait	Yes	145.8	142.7	148.9
Kyrgyzstan	No	166.5	162.2	170.8
Kyrgyzstan	Yes	169.3	159.7	178.8
Lao People's Democratic Republic	No	162.0	153.8	170.1
Lao People's Democratic Republic	Yes	164.8	143.8	185.8
Lebanon	No	149.5	144.6	154.4
Lebanon	Yes	152.9	144.4	161.3
Lesotho	No	157.0	151.5	162.6
Lesotho	Yes	161.5	151.7	171.2
Liberia	No	166.3	161.2	171.4
Liberia	Yes	165.6	147.4	183.8
Libya	No	169.1	164.1	174.1
Libya	Yes	176.3	169.7	182.9
Malawi	No	155.0	150.0	159.9
Malawi	Yes	140.5	133.0	148.0
Mongolia	No	151.5	149.5	153.5
Mongolia	Yes	155.0	149.7	160.3
Morocco	No	155.1	151.6	158.7
Morocco	Yes	165.6	161.1	170.1
Myanmar	No	154.7	152.4	157.1
Myanmar	Yes	150.8	144.9	156.8
Nepal	No	153.2	147.8	158.5
Nepal	Yes	154.4	145.0	163.9

Niue	No	157.5	150.4	164.7
Niue	Yes	148.4	142.5	154.3
Qatar	No	152.1	148.2	156.1
Qatar	Yes	147.1	144.2	149.9
Republic of Moldova	No	163.9	161.0	166.9
Republic of Moldova	Yes	157.9	154.5	161.4
Sao Tome and Principe	No	157.1	152.1	162.0
Sao Tome and Principe	Yes	150.0	140.5	159.6
Seychelles	No	151.9	149.6	154.2
Seychelles	Yes	152.2	147.5	156.9
Sri Lanka	No	155.2	152.1	158.3
Sri Lanka	Yes	158.6	154.7	162.6
Sudan	No	158.1	155.0	161.3
Sudan	Yes	158.2	152.2	164.3
Tajikistan	No	158.2	154.7	161.6
Tajikistan	Yes	173.9	159.8	187.9
Timor-Leste	No	159.8	155.6	164.0
Timor-Leste	Yes	168.8	162.6	175.0
Tokelau	No	148.9	144.5	153.4
Tokelau	Yes	152.0	142.6	161.3
Turkmenistan	No	155.5	152.7	158.2
Turkmenistan	Yes	159.1	152.6	165.6
Tuvalu	No	153.8	145.7	162.0
Tuvalu	Yes	170.2	156.3	184.1
Uganda	No	161.3	152.9	169.6
Uganda	Yes	149.3	128.7	169.9
United Republic of Tanzania	No	170.7	164.9	176.6
United Republic of Tanzania	Yes	159.7	146.3	173.1
Vanuatu	No	159.9	153.6	166.2
Vanuatu	Yes	157.4	148.5	166.3
Vietnam	No	152.1	148.3	155.9
Vietnam	Yes	147.9	139.5	156.3
Zambia	No	154.6	150.7	158.4
Zambia	Yes	163.9	149.8	178.0

CI: confidence interval.

Supplementary Table 9. Absolute and crude number of observations with self-reported hypertension and whose blood pressure was >130/80 mmHg by country.

Country	Total sample (n)	>130/80 mmHg (n)
Afghanistan	412	335
Algeria	558	428
American Samoa	151	135
Armenia	218	198
Azerbaijan	390	356
Bangladesh	562	450
Belarus	1253	1177
Benin	142	122
Bhutan	409	362
Botswana	435	358
Brunei Darussalam	353	280
Cabo Verde	129	105
Cambodia	237	139
Comoros	250	207
Cook Islands	108	86
Ecuador	351	225
Eritrea	142	116
Eswatini	232	208
Ethiopia	126	103
Fiji	247	227
Georgia	755	653
Guyana	148	107
Iraq	142	118
Jordan	195	139
Kenya	108	97
Kuwait	382	250
Kyrgyzstan	311	300
Lao People's Democratic Republic	92	76
Lebanon	201	132
Lesotho	190	170
Liberia	112	92
Libya	218	207
Malawi	138	109
Mongolia	1121	795
Morocco	288	251
Myanmar	843	707

Nepal	194	172
Niue	99	76
Qatar	311	229
Republic of Moldova	685	653
Sao Tome and Principe	169	152
Seychelles	362	323
Sri Lanka	459	383
Sudan	370	338
Tajikistan	257	245
Timor-Leste	109	87
Tokelau	51	45
Tonga	322	280
Turkmenistan	428	405
Tuvalu	77	74
Uganda	76	60
United Republic of Tanzania	154	128
Vanuatu	97	88
Vietnam	197	155
Zambia	138	116

Supplementary Figure 1. Flowchart of study population (sample size [number of countries]).

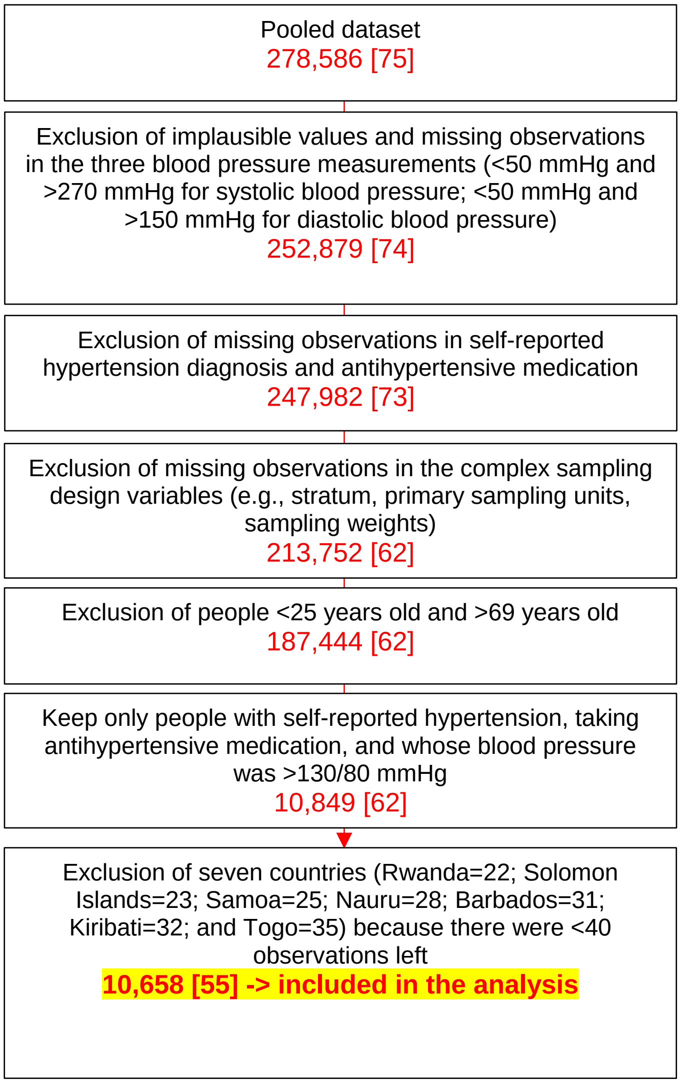

Supplementary Figure 2. Mean systolic blood pressure (mmHg) by country and grouped into world regions.

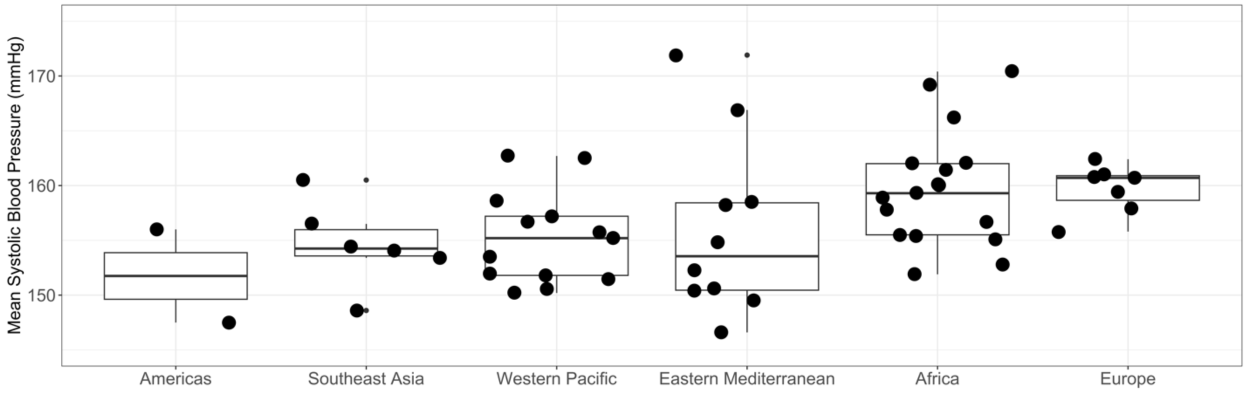

Each dot is a country.

Supplementary Figure 3. Mean systolic blood pressure (mmHg) by country and income group.

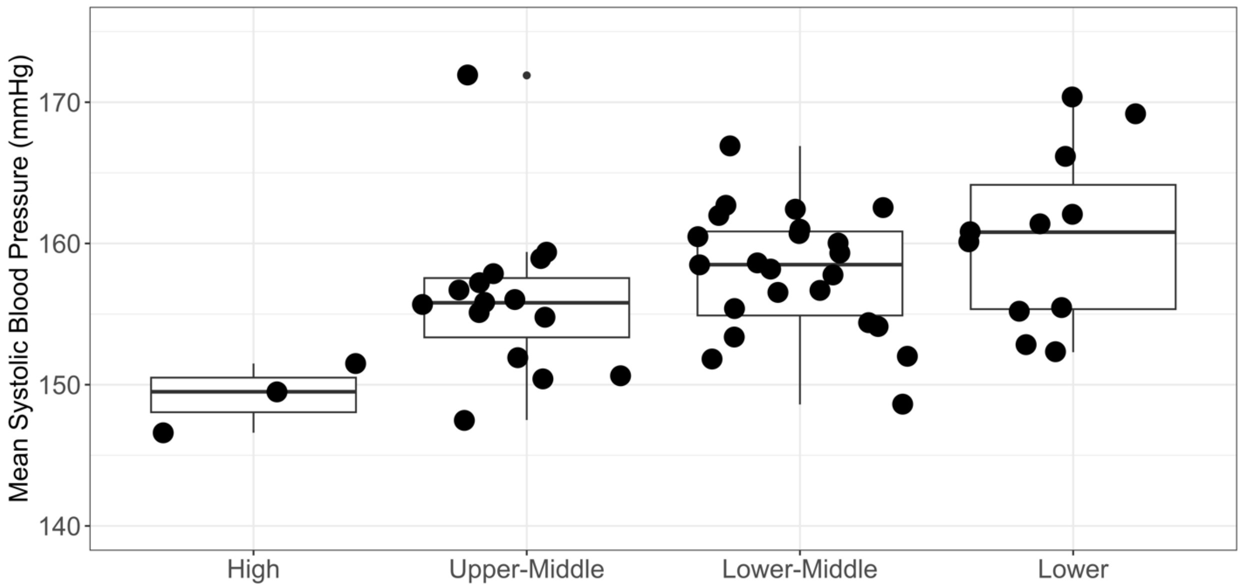

Each dot is a country.

Supplementary Figure 4. Mean systolic blood pressure (mmHg) by country between women and men and by world region.

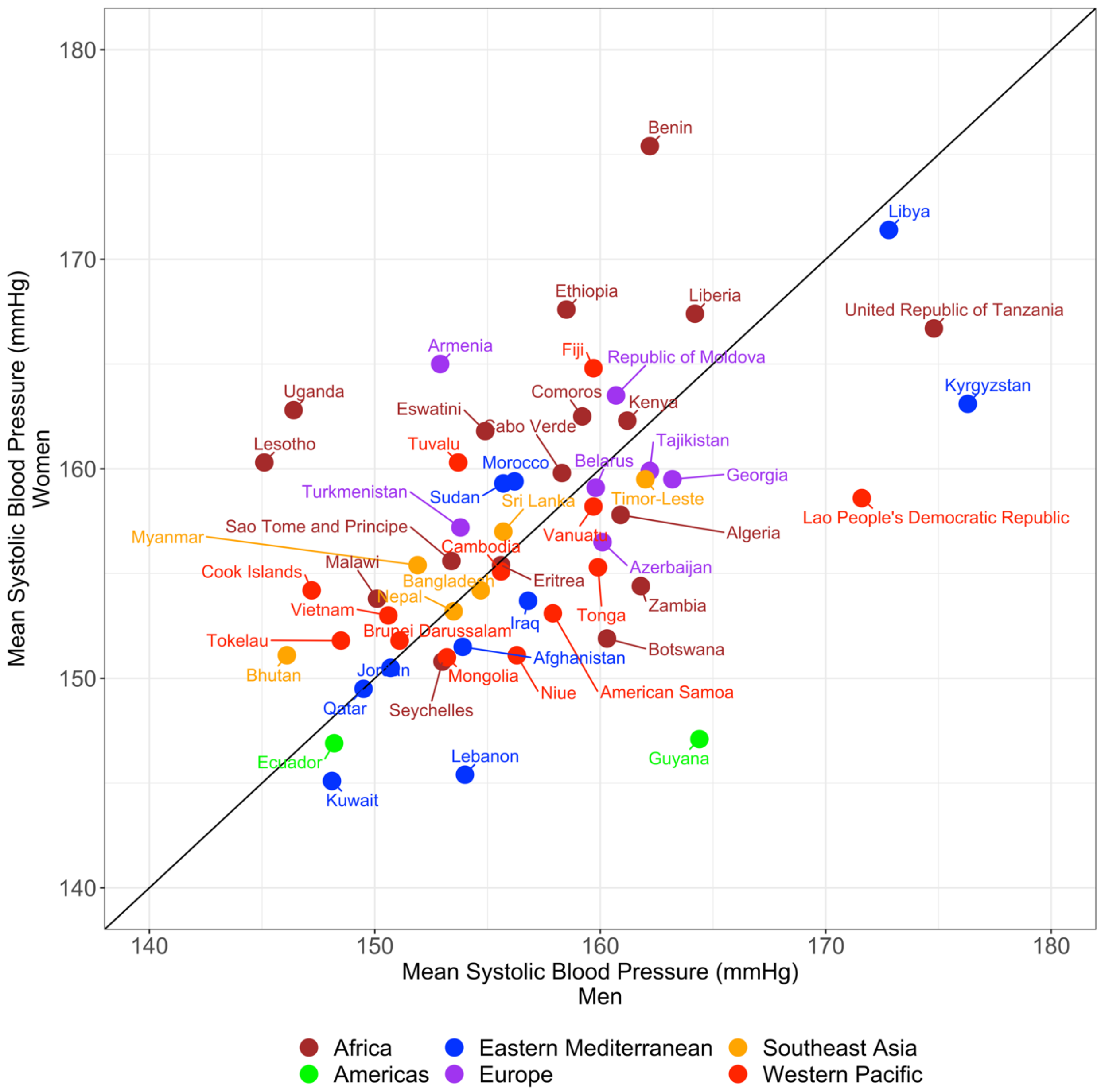

Each dot is a country.
